# DNA methylation analysis with nasal brushing for early diagnosis of sinonasal malignant tumours

**DOI:** 10.1007/s12672-026-04508-0

**Published:** 2026-01-29

**Authors:** Luca Morandi, Paolo Farneti, Anna Caterina Leucci, Giulia Querzoli, Sofia Melotti, Angela Camagni, Paolo Galli, Giacomo Sollini, Alessandro Franchi, Caterina Tonon, Raffaele Lodi, Ernesto Pasquini, Maria Pia Foschini

**Affiliations:** 1 Functional and Molecular Neuroimaging Unit, Bellaria Hospital, Department of Biomedical and Neuromotor Sciences, University of Bologna, via Altura 3, 40139 Bologna, Italy; 2 IRCCS Istituto delle Scienze Neurologiche di Bologna, Bologna, Italy; 3 Otorhinolaryngology Unit, Ospedale Bellaria Dip. Chirurgie Specialistiche, AUSL Bologna, Bologna, Italy; 4 Pathology Unit, IRCCS Azienda Ospedaliero-Universitaria di Bologna, Bologna, Italy; 5 Unit of Anatomic Pathology, AUSL Bologna, Maggiore Hospital, Bologna, Italy; 6 Unit of Anatomic Pathology, Department of Biomedical and Neuromotor Sciences, University of Bologna, Bologna, Italy; 7 Department of Innovation in Health and Social Services, Directorate-General for Health and Welfare, Emilia-Romagna Region, Bologna, Italy; 8 Department of Translational Research, Section of Pathology, University of Pisa, 56124 Pisa, Italy

**Keywords:** DNA methylation analysis, Epigenetic biomarkers, Sinonasal tumours, Sinonasal brushing, Early diagnosis

## Abstract

Sinonasal tumours are rare entities presenting with non-specific symptoms, therefore being often misinterpreted. The present study aimed to evaluate if the 13-gene DNA Methylation assay for early cancer detection already assessed in the oral cavity, was also useful in nasal cavity tumours. The case series consisted of 93 patients (63 males/30 females), 49 with malignant tumours, 14 with benign/borderline tumours, 34 as control series with 33 inflammatory polyps and one fungus ball. We collected one flocked swab from the lesion and one from the contralateral nasal cavity. All sinonasal cancer cases were evaluated by bisulfite next generation DNA sequencing, investigating the following genes: *ZAP70*,* ITGA4*,* KIF1A*,* PARP15*,* EPHX3*,* NTM*,* LRRTM1*,* FLI1*,* MIR193*,* LINC00599*,* MIR296*,* TERT*, *GP1BB*. To evaluate the performance of the methylation assay, a specific methylation score was calculated for each sample using linear discriminant analysis, with a predefined positivity threshold of 1.0615547. The association between diagnosis and methylation score positivity was evaluated through Fisher’s exact test and calculation of risk ratios with 95% confidence intervals. Additionally, dimensionality reduction techniques were employed to explore the structure of the dataset and assess the ability of methylation profiles to distinguish between different pathological conditions. The 13-gene DNA Methylation scored positive in 42/49 malignant tumours; We included also 14 benign/borderline tumours of which 11 scored positive. Among 33 inflammatory polyps, 24 scored negative, as well as 64/70 normal contralateral mucosa and one fungus ball. Therefore, excluding benign/borderline tumours, the detected sensitivity was 85.7% and specificity 85.6% (AUC: 0.878).

**Supplementary Information:**

The online version contains supplementary material available at 10.1007/s12672-026-04508-0.

## Introduction

Sinonasal malignant tumours are rare, accounting for only 0.5-1% of all malignancies in the Western population. They comprise 5% of all cancers of the head and neck region, with an annual incidence of approximately 1 case per 100.000 inhabitants worldwide [1]. Together, sinonasal squamous-cell carcinoma (SNSCC) and intestinal type adenocarcinoma (ITAC) account for 80% of all sinonasal tumours [2, 3]. They occur more commonly in adult-elderly men, with a male-to-female ratio of 2:1 in SNSCC and up to 6:1 in ITAC [4, 5].

The male predominance of sinonasal cancers is probably the result of the etiological involvement of occupational hazards. Sinonasal cancers tumorigenesis has been correlated to occupational exposure to several industrial compounds, the most frequent being wood and leather dust, in about 40% of all cases, 30% of SNSCC and 90% of ITAC specifically [6]. Exposure to such environmental insults usually begins at an early age and often persists for longer than 20 years. Professional wood workers have up to 500–900-times and 20-times increased risk of developing ITAC and SNSCC respectively, compared with the general population [7]. Apart from wood and leather dust, chemical substances such as glues, formaldehyde, chrome, nickel, and various compounds used in the textile industry have been associated with sinonasal carcinomas, mainly ITAC and SNSCC. Based on this evidence, in many European countries ITAC is officially considered a professional disease [8].

Early symptoms of nasal cavity cancer share same clinical presentation as many other more frequent lesions, as chronic rhinitis or sinusitis [3]. Owing to the nonspecific and the often relatively mild nature of the early symptoms, sinonasal malignancies have a prolonged diagnostic latency [9], frequently leading to discover tumours at advanced stage [10].

Early detection tools and adequate pre-operative diagnosis are needed to find out patients with early neoplastic lesions. The diagnosis of sinonasal tumour requires nasal endoscopy and biopsy sampling, procedures that can create discomfort and may be refused by the patient.

Therefore, the development of noninvasive methods for early detection is an attractive strategy to reduce the burden of nasal cavity carcinoma.

In the last decade, the interest in epigenetic mechanisms regulating tumor development has gained attention. DNA methylation is a common epigenetic mechanism leading to gene silencing in tumours. Specifically, DNA methylation refers to the covalent addition of a methyl group to the 5th carbon position of cytosine bases that are located 5′ to a guanosine base in a CpG dinucleotide. CpG dinucleotides are usually found clustered in specific regions, named CpG islands, which are often located in the promoter of several genes, including tumor suppressor genes and proto-oncogenes [11]. Aberrant DNA methylation in these loci may contribute to cancer progression, leading to dysregulation of mRNA expression, an early and frequent event in tumours. On the other side, DNA hypomethylation promotes tumorigenesis via transcriptional activation of oncogenes and chromosomal instability [12].

Previous studies, developed at our institution [13–18], demonstrated that the pre-operative evaluation of methylation profile of a panel of 13 genes (*ZAP70*,* ITGA4*,* KIF1A*,* PARP15*,* EPHX3*,* NTM*,* LRRTM1*,* FLI1*,* MIR193*,* LINC00599*,* MIR296*,* TERT*, and *GP1BB*), based on cell collection by brushing the lesion, was useful in discriminating benign from potentially malignant or malignant oral lesions.

The present study aimed to evaluate if the 13-gene DNA Methylation analysis already assessed in the oral cavity, is also useful to the early detection of nasal cavity tumours. For this purpose, a preliminary series of nasal cavity malignancies was evaluated applying the same 13-gene DNA Methylation approach.

## Materials and methods

### Study setting and data collection

All consecutive patients presenting at the Otolaryngology Unit at Bellaria Hospital, Bologna (Italy) from January 2022 to January 2024 with a mass of the sinonasal region, were included in this preliminary observational study. Selection criteria were as follows: patients aged > 18 years, presenting with a mass of the sinonasal region, suspicious for neoplasm, requiring incisional biopsy for diagnostic purposes.

Cases presenting with inflammatory polyps, candidate to surgical excision and histological analysis were included as negative control.

Traumatic lesions and all lesions that do not require histological examination for diagnosis were excluded.

Nasal brushing specimens were always picked before the incisional biopsy for histological diagnosis. Brushing was performed on both nasal cavities, the one with the neoplastic mass and the normal one.

Histological examination for the diagnosis of each lesion was performed on a blinded basis at the Unit of Anatomic Pathology at our Institute. All of the cases were examined by three pathologists (M.P.F., G.Q. and S.M.) with specific knowledge on head and neck tumours. Histological diagnoses were performed following the World Health Organization Head and Neck Tumor Classification, 5th Edition (WHO 2022) [19].

### Brushing

Nasal brushing was performed according to a previously described protocol [13], shortly summarized as follows: a flocked swab was used to collect exfoliated cells from nasal mucosa (SG-Nasal Collection Kit, Studium Genetics Srl, Bologna, Italy). In the neoplastic lesions the surface was gently brushed repeatedly five times. Brushing cell collection was always performed before incisional biopsy and without the use of any local anesthetic. The same procedure was performed in the middle meatus/olfactory fossa of the contralateral side. Brushing was always performed under direct visualization with a 0° nasal endoscope by three different experienced ENT surgeons (P.F., G.S. and E.P.). After brushing, each flocked swab was placed in a 1.5-mL tube containing a solution for nucleic acids preservation at room temperature.

### Analytic strategy

A comprehensive descriptive of data was provided. A multi-step analytical strategy was carried out to ensure a comprehensive evaluation of DNA methylation alterations and their clinical significance in sinonasal tumours. By combining DNA methylation profiling, dimensionality reduction techniques and statistical modeling, this study allows to: (i) differentiate malignant sinonasal tumours from benign conditions using epigenetic markers; (ii) evaluate the diagnostic reliability of the 13-gene methylation assay as a non-invasive early detection tool; (iii) identify potential pre-malignant alterations in benign/borderline lesions, providing insights into sinonasal carcinogenesis.

### DNA methylation analysis

DNA methylation analysis was performed as previously described by Morandi et al. [13]. Briefly, DNA from exfoliated cells was purified using the Quick DNA MagBead Plus kit (cat. no. D4081; Zymo Research, Irvine, CA, USA) and were treated with sodium bisulfite using EZ-96 DNA Methylation MagPrep (cat. no. D5041; Zymo Research) according to the manufacturer’s instructions. Quantitative DNA methylation analysis was performed by next-generation sequencing for the following genes: *ZAP70*,* ITGA4*,* KIF1A*,* PARP15*,* EPHX3*,* NTM*,* LRRTM1*,* FLI1*,* MIR193*,* LINC00599*,* MIR296*,* TERT*,* GP1BB*, and *H19* as an imprinted gene as a control. The regions of interest were described elsewhere [13]. Libraries were prepared using the Nextera™ Index Kit (Illumina, San Diego, CA, USA, FC-121-1012) following a two-steps PCR approach and loaded onto MiSEQ (Illumina, San Diego, CA, USA, cod. 15027617). Each NGS experiment was designed to allocate at least 1000 reads/amplicon to reach a depth of coverage of at least 1000×. FASTQ output files were evaluated for quality control (> Q30), processed by BWAmeth and by MethylDackel to generate .bam and .bai and excel files respectively in a Galaxy Project environment [20]. In our previous study [13], the best CpGs identified by receiver operating characteristics (ROC) analysis were used in a linear discriminant analysis to develop the algorithm. The final score was able to identify Oral Squamous Cell Carcinoma (OSCC) with a threshold of 1.0615547 as the best value for sensitivity and specificity (AUC = 0.981). Using the same algorithm, in this study, we calculated the specific score for each sinonasal sample. Values exceeding the threshold of 1.0615547 were considered positive. Methylation plotter tool was used to compare DNA methylation level of the 13 gene panel and clinicopathological variables [21] (see Supplementary File 1). ClustVis, a web tool for visualizing clustering multivariate data (http://biit.cs.ut.ee/clustvis/) [22] was used to generate graphical representation of methylation level at single CpG position (see supplementary File 2). The original sequencing data presented in the study are openly available in the NCBI Sequence Repository Archive (SRA) at PRJNA1322681.

### Multivariate data projection methods

To explore the structure of the data and identify potential clinical patterns, we employed two different dimensionality reduction techniques: Principal Component Analysis (PCA), Uniform Manifold Approximation and Projection (UMAP). Each of these methods provides unique insights into the dataset and allows us to validate the robustness of our findings.

PCA is a linear transformation technique that identifies the directions (principal components) in which the variance of the data is maximized. It is particularly useful for understanding the overall variance structure and simplifying the dataset while preserving most of its variability.

UMAP is a non-linear dimensionality reduction method that excels in preserving both local and global structures within the data. Unlike PCA, which is strictly linear, UMAP is designed to capture complex relationships and provide a meaningful visualization of high-dimensional datasets.

By applying different methods, we ensured a comprehensive analysis of the dataset. PCA provided a clear view of variance distribution, UMAP captured both global and local structures.

### Methylation score

Finally, a specific score for each sample was elaborated using linear discriminant analysis. Values exceeding the threshold of 1.0615547 were considered positive, as previously described for oral squamous cell carcinoma [13]. To validate the score’s ability to detect malignancies, this part of the analysis first assessed sensitivity using a ROC-curve analysis then focused on describing the score stratified by diagnosis and estimating the differences in the probability of obtaining a positive result across diagnoses. To assess the association between a positive methylation score (1 if score ≥ 1.0615547, 0 otherwise) and specific diagnostic groups, we computed risk ratio along with 95% confidence intervals (CIs), using the group with normal nasal mucosa as the reference category. The relative risk quantifies how much more likely it is for a positive methylation result to occur in each diagnostic group compared to normal controls. Additionally, Fisher’s exact test was employed to assess the statistical significance of the association between diagnosis and methylation score positivity. This test was chosen due to the limited sample size in several subgroups, ensuring robust evaluation of categorical differences without relying on large-sample approximations.

## Results

Results are summarized in Table 1. This preliminary study included a case series consisting of a dataset of 93 patients (63 males and 30 females) with a mean age at diagnosis of 61 years (range 18–86 years). Forty-nine cases were malignant tumours (4 cases were bilateral), 14 with benign/borderline tumours, 34 patients as control series with 33 inflammatory polyps and one fungus ball.

**Table 1:** Results according to histology

Tumour type	*N*. of cases	Positive score	Negative score
Malignant tumours (main site)	**49**	42 (85,71%)	7 (14,29%)
ITAC	23	21 (90,48%)	2 (9,52%)
Adenocarcinoma of non-intestinal type	1	0 (0%)	1 (100%)
Keratinizing squamous cell carcinoma	6	6 (100%)	0 (0%)
Non-keratinizing EBV-related carcinoma	1	0 (0%)	1 (100%)
Nonkeratinizing, lymphoepithelioma-like squamous cell carcinoma	1	1(100%)	0(0%)
Mucosal melanoma	4	4 (100%)	0 (0%)
Neuroblastoma	2	1 (50%)	1 (50%)
Adenoid cystic carcinoma	1	1 (100%)	0 (0%)
Teratocarcinosarcoma	1	1 (100%)	0 (0%)
Rhabdomyosarcoma	1	1 (100%)	0 (0%)
Alveolar rhabdomyosarcoma	1	1 (100%)	0 (0%)
Solitary fibrous tumour with dedifferentiated areas	1	0 (0%)	1 (100%)
Ewing sarcoma	1	1 (100%)	0 (0%)
Anaplastic lymphoma	1	1 (100%)	0 (0%)
Diffuse large B cell lymphoma	1	0 (0%)	1 (100%)
Sinonasal undifferentiated carcinoma	1	1 (100%)	0 (0%)
SWI/SNF complex deficient sinonasal carcinoma	1	1 (100%)	0 (0%)
Papillary squamous cell carcinoma	1	1 (100%)	0 (0%)
Benign/borderline tumours	14	11 (72.72%)	3 (27,27%)
Sinonasal papilloma, inverted type (2 with HG Dysplasia)	11	10 (90%)	1 (10%)
Sinonasal papilloma, oncocytic type	1	1 (100%)	0 (0%)
Glomangiopercytoma	1	0 (0%)	1 (100%)
Ectopic pituitary neuroendocrine tumour	1	0 (0%)	1 (100%)
Inflammatory polyps	**33**	**9 (37.50%)**	**24 (62.50%)**
Normal contralateral mucosa	**70**	**6 (8.57%)**	**64 (91.43%)**
Fungus ball	**1**	**0 (0%)**	**1 (100%)**

*ITAC* intestinal type adenocarcinoma

Malignant tumours were diagnosed as follows: adenocarcinoma of intestinal type (ITAC, 23 cases), adenocarcinoma of non-intestinal type (1 case), keratinizing SNSCC (6 cases), non-keratinizing Epstein-Barr Virus (EBV)-related lymphoepithelioma-like SNSCC (2 cases), mucosal melanoma (4 cases), olfactory neuroblastoma (2 cases), adenoid cystic carcinoma (1 case), teratocarcinosarcoma (1 case), rhabdomyosarcoma (1 case), alveolar rhabdomyosarcoma (1 case), solitary fibrous tumour with dedifferentiated areas (1 case), Ewing sarcoma (1 case), anaplastic lymphoma (1 case), diffuse large B cell lymphoma (1 case), sinonasal undifferentiated carcinoma (1 case), SWI/SNF complex deficient sinonasal carcinoma (1 case) and a papillary SNSCC (1 case). Three out of 23 cases of ITAC and one out of 6 cases of keratinizing SNSCC were bilateral.

In addition, 11 cases of sinonasal papilloma inverted type (SNPI), one of which with high-grade dysplasia, one case of sinonasal papilloma oncocytic type, one case of glomangiopericytoma and one case of ectopic pituitary neuroendocrine tumour (PitNet) were included as benign/borderline tumour (total number: 14).

Overall, the control series consisted of 33 patients with inflammatory polyps, 20 males and 13 females, with a mean age at diagnosis of 53 *(range 24–75)*, one fungus ball and 70 contralateral normal mucosa. Among all of 104 control cases, 89 were detected negative as expected and only 15 exceeded the threshold (specificity: 85.6%). In all patients with malignant and benign tumours, one flocked swab from the side of the lesion and one from the contralateral nasal cavity were collected. Additionally contralateral normal cases in only 15 inflammatory polyps served as controls.

For simplicity for statistical analysis, the following classes were identified: (1) SNSCC which also included mucosal melanoma, neuroblastoma, adenoid cystic carcinoma, teratocarcinosarcoma, rhabdomyosarcomas, solitary fibrous tumour, sarcomas, lymphomas; (2) ITAC; (3) SNPI/benign; (4) Inflammatory Polyps; (5) Normal.

The 13-gene DNA Methylation scored positive in 42/49 affected sides (sensitivity: 85.7%). The false negative cases consisted of: 2 out of 23 ITAC (9.52%), the adenocarcinoma of non-intestinal type, the non-keratinizing EBV-related carcinoma, 1 out of 2 neuroblastomas (50%), the solitary fibrous tumour with dedifferentiated areas and the diffuse large B cell lymphoma.

All the remaining malignant tumours scored positive, including cases of non-epithelial malignancies (mucosal melanoma, rhabdomyosarcoma, Ewing sarcoma and anaplastic lymphoma).

The 13-gene DNA Methylation scored positive in 11/14 benign/borderline lesions (72.72%).

Ten out of 11 cases of SNPI scored positive (90.90%), including one case showing high-grade dysplasia. Surprisingly, even the oncocytic papilloma scored positive, while glomangiopericytoma and pituitary ectopic adenoma, scored negative.

The 13-gene DNA Methylation scored negative in 64 out of 70 (91.43%) cases of normal contralateral mucosa from patients with benign/malignant tumours and inflammatory polyps. The false-positive cases were related to the contralateral nasal mucosa of one patient with mucosal melanoma, two patients with SNPI, one patient with adenoid cystic, one patient with alveolar rhabdomyosarcoma and a patient with inflammatory polyp.

**Fig. 1:**
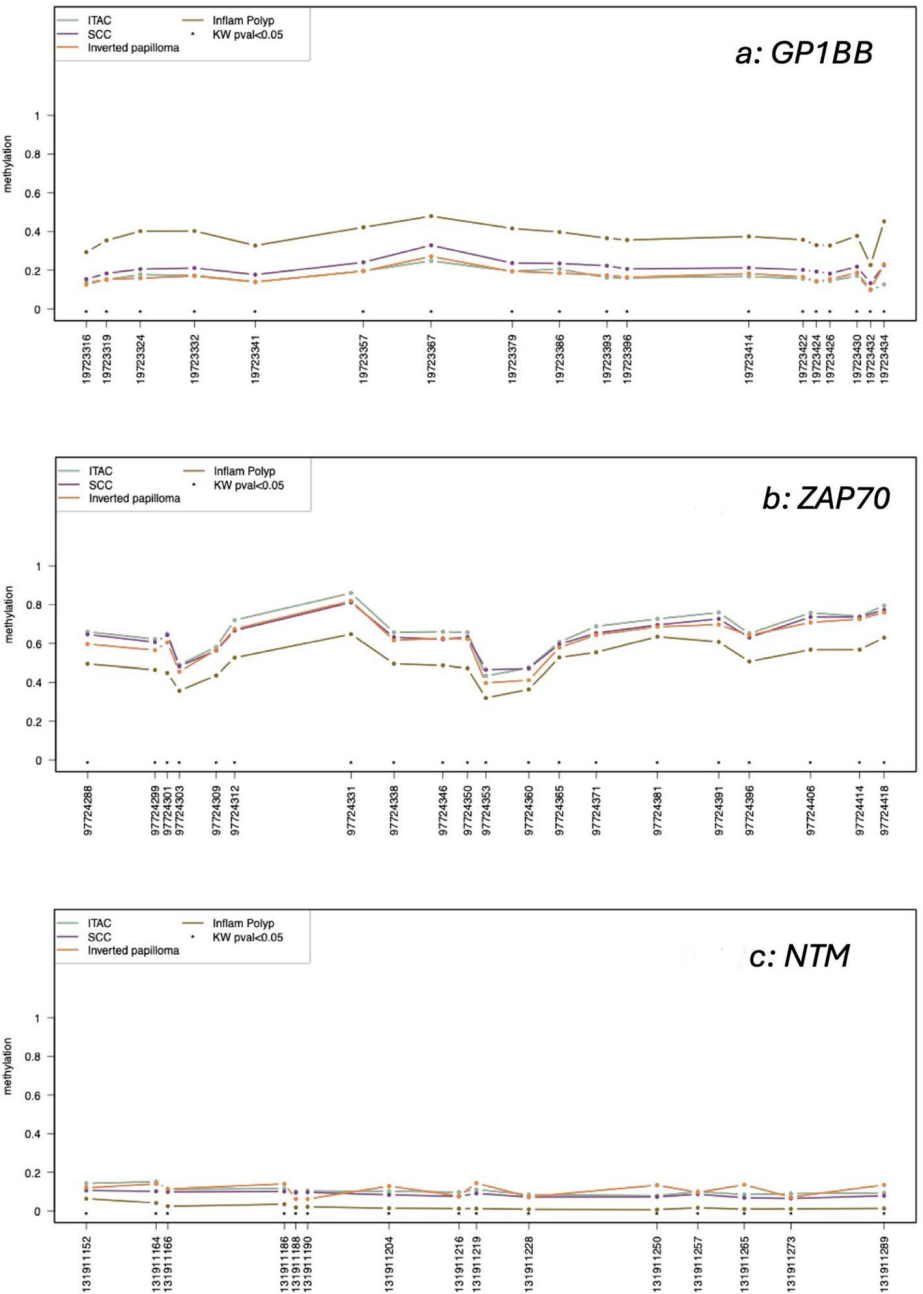
Methylation profile plot from *GP1BB* (**a**), *ZAP70* (**b**) and *NTM* (**c**). For each group of samples, each line represents the methylation mean for each position. Asterisks indicate a statistical significance as calculated by the Kruskal-Wallis test. *ZAP70* and *GP1BB*, together with *NTM* (see Supplementary File 1 for the methylation profile of all targets), revealed a fluctuating behavior among the various CpGs evaluated. The gap between Inflammatory polyps with respect to SCC, ITAC and inverted papilloma remained mostly the same (Kruskal-Wallis P values were < 0.05)

Regarding the investigated 13 genes, *GP1BB*,* TERT* and *MIR296* were detected as hypomethylated in malignant cases, while *ZAP70*,* ITGA4*,* KIF1A*,* PARP15*,* NTM*,* MIR193*,* LRRTM1* and *EPHX3* were found hypermethylated in tumours, following the same pattern of oral squamous cell carcinoma. The best discriminatory performances were obtained for *GP1BB* with all 18 out of 18 investigated CpG which were statistically significant for Kruskal Wallis test (Fig. 1a), *ZAP70* with 20/20 (Fig. 1b), *NTM* with 15/15 (see Fig. 1c), followed by *MIR193* with 17/26 and *PARP15* with 3/19. *EPHX3* clustered SNSCC with ITAC (hypermethylated) and SNPI with inflammatory polyps (hypomethylated). Low level of methylation in *KIF1A* was detected only in SNSCC, ITAC and SNIP. *ITGA4* revealed hypermethylation in SNSCC, ITAC and partially in inflammatory polyps, while SNIP was totally hypomethylated. *FLI1* is hypermethylated only in ITAC and SNSCC. With respect to *LINC0599*, hypermethylation was found only in ITAC and SNPI, not in SNSCC. *LRRTM1* showed hypermethylation only in SNIP, ITAC, and only partially in SNSCC. *MIR296* revealed moderate hypomethylation only in ITAC and *TERT* only in SNSCC. Regarding SNIP, we detected a peculiar behavior in *ITGA4* (hypomethylation), *LINC00599*,* LRRTM1 and MIR193* (hypermethylation). This allows us to stratify this group from the others. All methylation profile plots, box plot, and descriptive analysis of each gene is available in Supplementary File 1.

The methylation plots of each gene are available in Supplementary File1). The HeatMap, using correlation distance and average linkage with all the CpG investigated, pointed out two different groups: a right cluster showing 54 normal samples, 19 inflammatory polyps, 6 tumours, 5 ITAC and 3 inverted papilloma; left cluster shows 20 tumours, 18 ITAC, 11 inverted papilloma, 14 inflammatory polyps and 17 normal cases (see Supplementary File 2).

### Multivariate data projection results

**Fig. 2:**
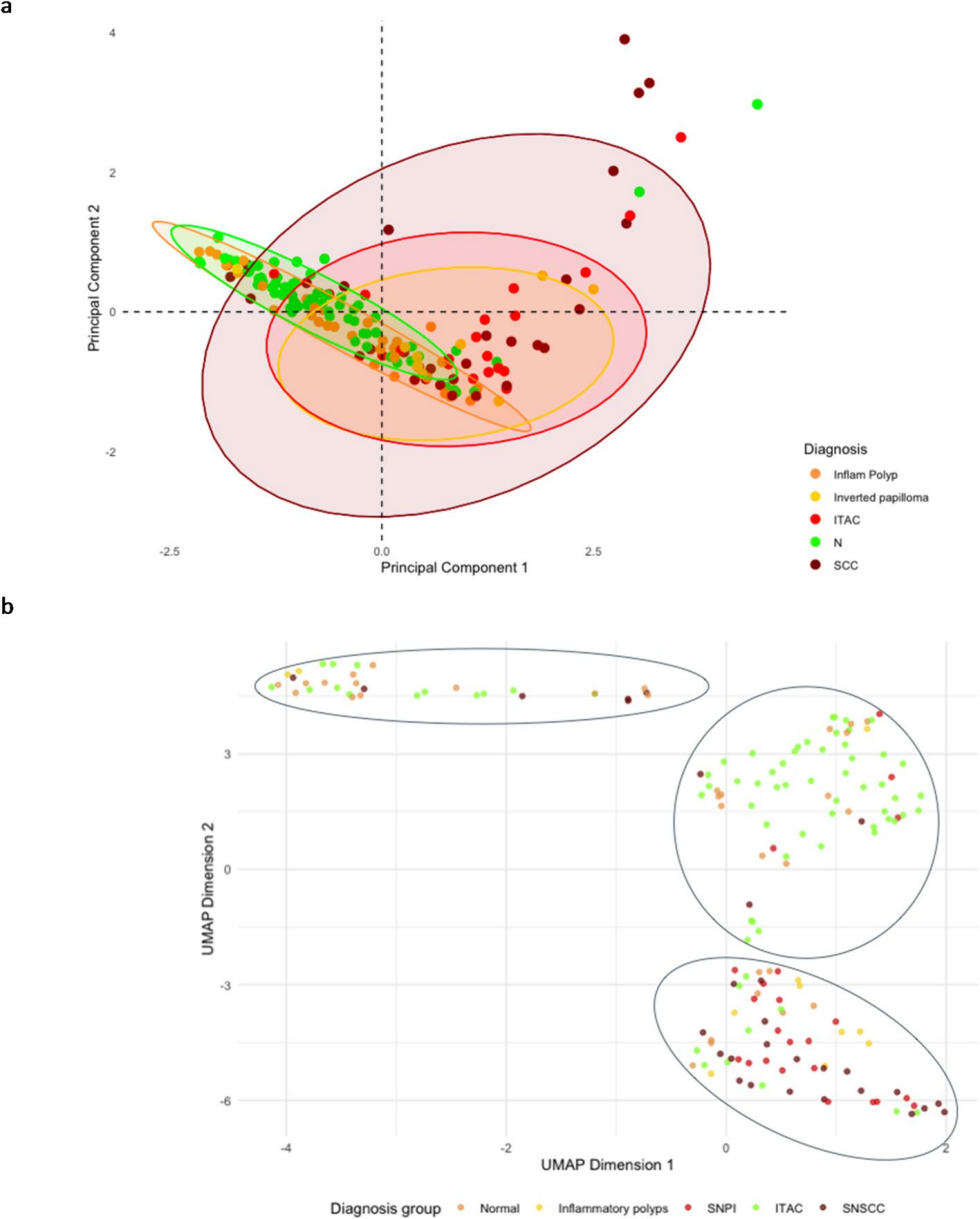
PCA results: distribution of samples based on PCA (**a**). Malignant tumours, benign lesions, and inflammatory conditions are plotted along the first two principal components, with malignant cases showing greater variability. PCA provides a global view of variance. UMAP visualization of the dataset highlighting clear separation between malignant tumours, benign lesions, and inflammatory conditions (**b**)

PCA revealed distinct clustering among different sample groups, with malignant tumours, ITAC, and inflammatory polyps forming separate clusters (Fig. 2a). Specifically, PCA showed that malignant samples exhibited greater variability, whereas benign conditions, such as inflammatory polyps, clustered more tightly together, indicating lower epigenetic diversity.

UMAP further confirmed these separations, showing a well-defined distinction between malignant and benign conditions (Fig. 2b). The malignant tumours displayed a wider distribution, while ITAC showed a partial overlap with the other malignant group, suggesting some shared epigenetic alterations. Furthermore, UMAP visualization emphasized the heterogeneity within the malignant group, indicating potential subtypes with varying degrees of epigenetic dysregulation.

### Methylation score results

**Fig. 3:**
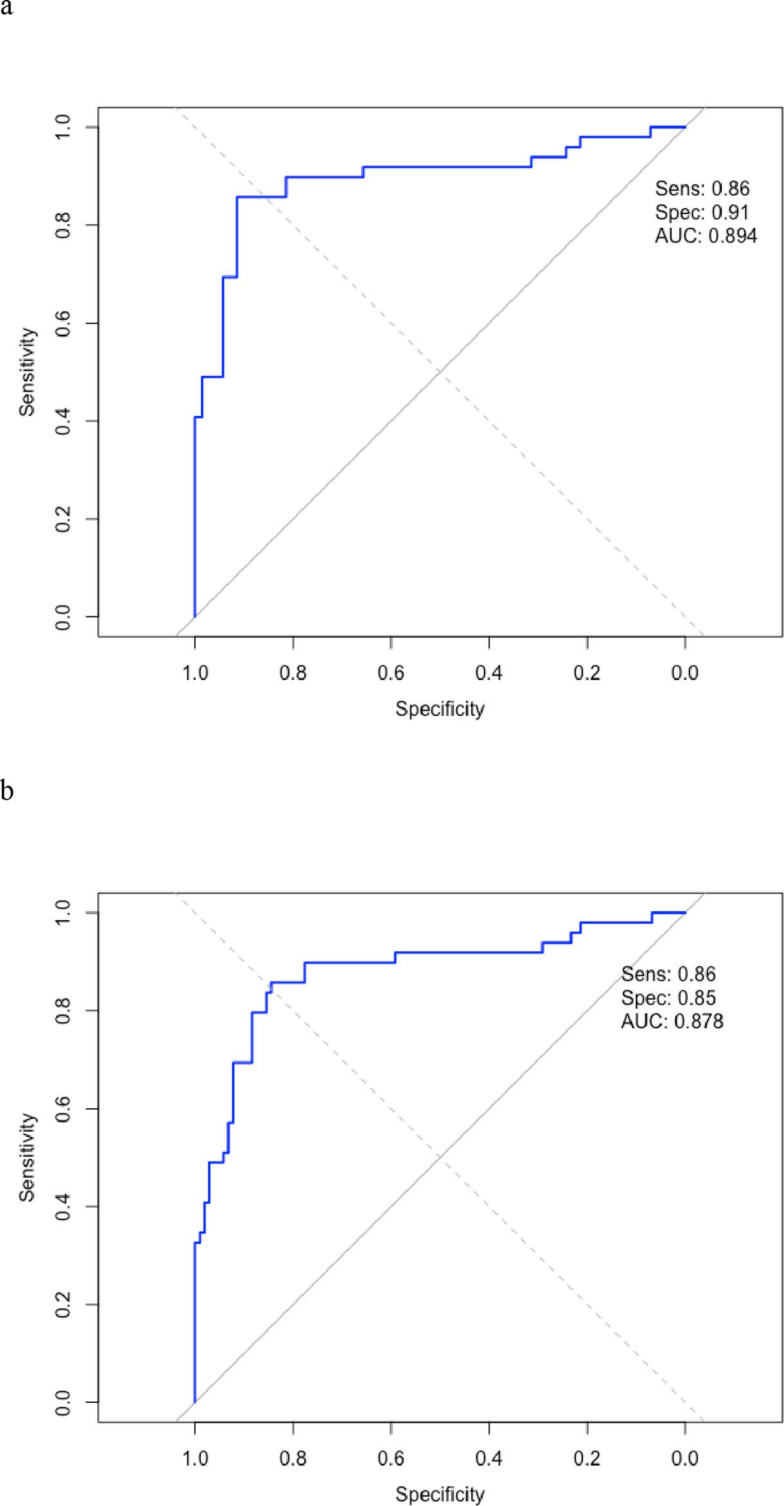
ROC curve in (**a**) was constructed considering malignant tumours (*n* = 49) versus contra-lateral normal nasal mucosa only (*n* = 70). At the predefined threshold (methylation score ≥ 1.0615547), the assay yielded a sensitivity of 0.86 and a specificity of 0.91. In (**b**), the control group was expanded to include both contralateral normal mucosa (*n* = 70) and inflammatory polyps (*n* = 33), for a total of 103 negative cases (sensitivity: 0.86; specificity: 0.85)

ROC curve analysis (Fig. 3) was conducted to assess the diagnostic performance of the 13-gene DNA methylation score in differentiating malignant sinonasal tumours from non-malignant conditions. In Fig. 3a, the ROC curve was constructed considering malignant tumours (*n* = 49) versus contra-lateral normal nasal mucosa only (*n* = 70). At the predefined threshold (methylation score ≥ 1.0615547), the assay yielded a sensitivity of 0.86 and a specificity of 0.91. In Fig. 3b, the control group was expanded to include both contralateral normal mucosa (*n* = 70) and inflammatory polyps (*n* = 33), for a total of 103 negative cases. Under these conditions, sensitivity remained at 0.86, while specificity slightly decreased to 0.85, reflecting the higher rate of methylation positivity among inflammatory polyps. Both curves display a steep initial ascent and approach the top-left corner of the plot, indicating strong overall diagnostic performance. The high AUC and consistent sensitivity further support the stability of the methylation score as a classifier. The slight decrease in specificity when including inflammatory polyps is visually reflected by a minor shift of the curve away from the top-left corner, suggesting a degree of epigenetic overlap in inflamed but non-malignant tissue. These findings support the robustness of the methylation score as a diagnostic tool, while also highlighting the potential for false-positive results in chronically inflamed but non-neoplastic mucosa.

**Fig. 4:**
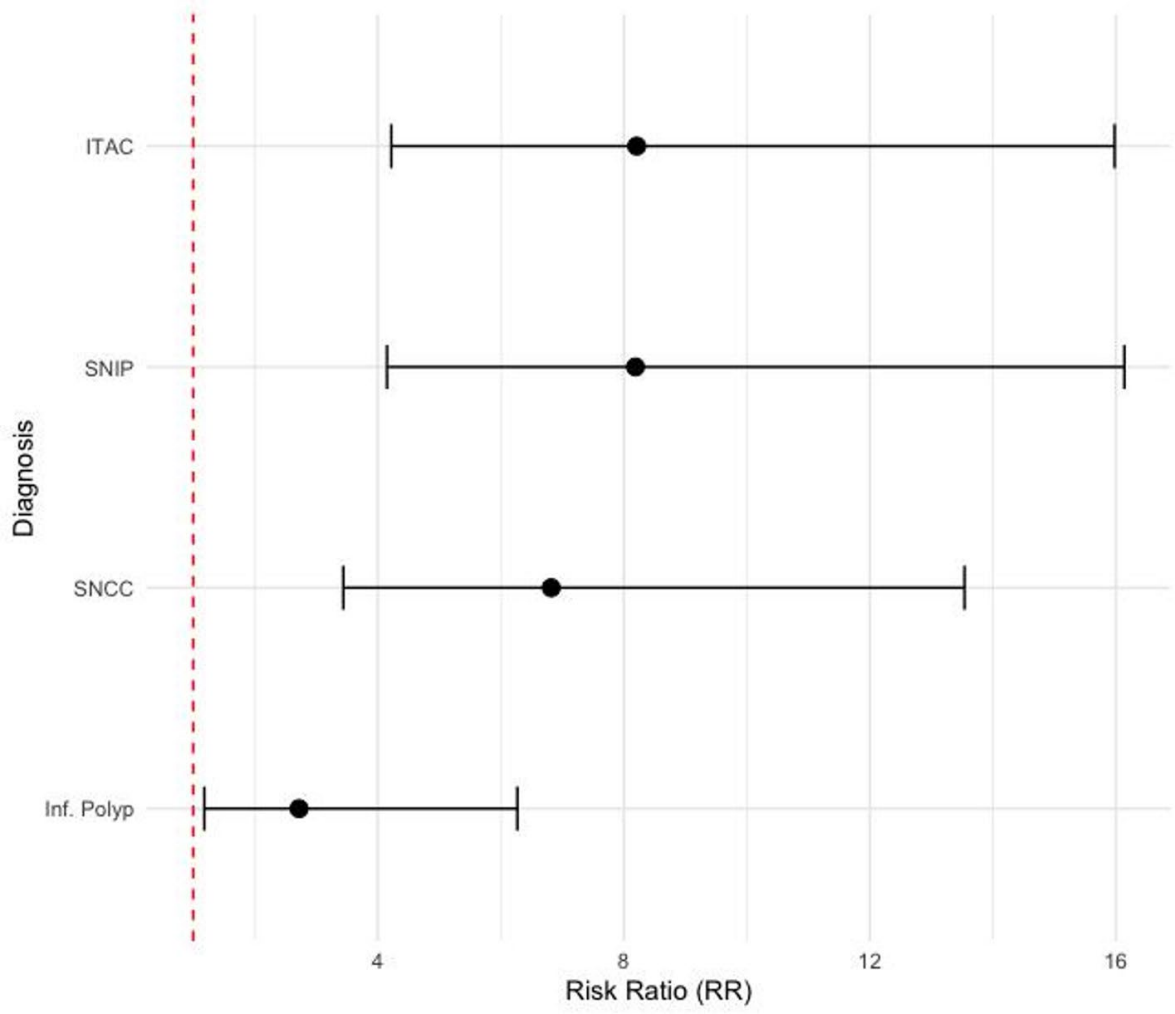
Forest plot showing relative risks and 95% CIs for methylation score positivity across different diagnostic groups, using normal mucosa as the reference. The red dashed vertical line represents the null value (Risk Ratio = 1), indicating no increased risk. Values to the right of this line indicate an elevated likelihood of a positive methylation result compared to normal tissue

The risk of obtaining a positive methylation score was significantly higher in all groups compared to the normal mucosa. In particular, the SNIP group showed the highest relative risk (RR = 8.19, 95% CI: 4.15–16.14, *p* < 0.0001), comparable to those observed in ITAC (RR = 8.21, 95% CI 4.22–15.98) and SNSCC (RR = 6.82, 95% CI 3.44–13.54). Inflammatory polyps also showed a significantly increased relative risk (RR = 2.72, 95% CI 1.18–6.27), although to a lesser extent (Fig. 4 and Supplementary File 3). These findings support the strong association between methylation positivity and neoplastic or pre-neoplastic lesions. They also confirm the high discriminatory power of the methylation score in distinguishing malignant from benign conditions. We did not observe any clear trend or statistically significant correlation between the quantitative value of the methylation score and either T-classification or nodal status. The same findings were observed with OSCC.

## Discussion

The need of a simple and reliable tool to obtain an early diagnosis of sinonasal carcinomas, led us to test the 13-gene DNA Methylation method in a series of benign and malignant sinonasal tumours.

Overall, an aberrant methylation pattern was detected in all evaluated genes in the present cohort, with 10 hypermethylated and three hypomethylated genes found in malignant tumours and ITAC. The same epigenetic aberrations were found in oral squamous cell carcinoma.

Our results showed a sensitivity of 85.7% and specificity 91.43% considering normal contralateral mucosa as a normal reference. False negative scores could be the result of sub-optimal brushing, not reaching the lesion. False positives scores in 9/24 inflammatory polyps might be the consequence of alterations at the methylation level of DNA due to inflammatory conditions. In fact, if we considered also inflammatory polyps as negative controls, the specificity slightly decreased to 85.6%, highlighting a minor shift of the curve away from the top-left corner, suggesting a degree of epigenetic overlap in inflamed but non-malignant tissue. In any case, the high AUC and consistent sensitivity support the stability of the methylation score as a classifier.

Moreover, the inflammatory polyps scoring positive presented erosions on the superficial mucosa with regenerative features of the epithelium or contextual severe chronic rhinosinusitis.

The possible sample contamination with few cancer cells was excluded, as the 13-gene DNA-methylation algorithm is based on quantitative methylation analysis and not simply on the methylated/unmethylated status. Therefore it’s quite difficult that few cancer cells could have distorted the result. The positive score detected in contralateral mucosa may be derived by the effect of “field cancerization” phenomenon that was firstly introduced by Slaughter in 1953 [23]. It was used to describe the early changes to the epithelium resulting from carcinogens and environmental agents that lead to multifocal tumours development in the oral cavity [24]. Cells with this kind of genetic and epigenetic changes gain the ability to develop and expand the neoplastic field and can be considered pre-cancerous cells [25]. Since then, the field cancerization concept has been applied in several organs to explain the occurrence of multiple primary cancers, among which the sinonasal region is comprised [26].

Quite unexpectedly, ten out of 11 cases of SNPI scored positive at the 13-gene DNA-Methylation assay. SNPI is defined a benign epithelial neoplasm, often associated with smoke, exposure to occupational and/or industrial substances and or to high-risk HPV (WHO 2022) [19]. Malignancies (the most frequent being keratinizing SNSCC) can develop SNPI in 2–4% of cases [27]. Indeed, one of the cases that scored positive, presented high-grade dysplasia of the epithelium. Mu et al. reported recently four up-regulated methylation genes (*UCKL1*,* GSTT1*,* HLA-G*,* MAML2*) and one down-regulated gene (*NRGN)* as indicators to stratify the malignant transformation potential of SNPI [28].

A peculiar pattern of SNIP detected in *ITGA4*,* LINC00599* and *LRRTM1*, allow us to stratify this group from the others.

An interesting point emerging from the present series is the cross-sectionality in application for epithelial and non-epithelial neoplasms, encompassing mesenchymal, melanocytic, lymphoid, and neuroectodermal neoplasms among them. This probably depends on sharing some epigenetic alterations in development and progression by these neoplastic lesions. For example, *LINC00599*, also known as *MIR124-HG*, has been found to be aberrantly methylated in breast and gastric cancer [29, 30]; *ITGA4* encodes a member of the integrin alpha chain family functioning in cell surface adhesion and signaling; it has been shown to be aberrantly altered in colorectal tumor and other malignancies [31]; *ZAP70* encodes a tyrosine kinase normally expressed by natural killer cells and T cells and its hypermethylation could be correlated to the immune microenvironment and can also predict an unfavorable disease course in terms of disease progression and overall survival in chronic lymphocytic leukemia [32]. In addition, *TERT* encoding telomerase has been found to be hypomethylated in different neoplasms [33, 34], while *EPHX3* [35], *NTM* [36], *FLI1* [37], *PARP15* [38] are usually found hypermethylated in several carcinomas.

Weakness of this study is the limited number of samples related to rare entities such as adenocarcinoma of non-intestinal type, non-keratinizing EBV-related lymphoepithelioma-like SNSCC carcinoma, olfactory neuroblastoma, adenoid cystic carcinoma, teratocarcinosarcoma, rhabdomyosarcoma, solitary fibrous tumour, sarcoma, lymphoma, diffuse large B cell lymphoma, sinonasal undifferentiated carcinoma, SWI/SNF complex deficient sinonasal carcinoma or papillary SNSCC. However, the ability of the algorithm to adequately discriminate malignant lesions in general with respect to inflammatory or normal mucosa, will allow the introduction of the test in the clinical setting, as it is non-invasive.

Brushing in this study has been performed under direct visualization with 0° nasal endoscope in order to better visualize the lesion and to obtain a proper sample both on the affected and in the healthy side of the nose. However, once the validity of this method has been established, it could be applied to patients even without direct endoscopic monitoring. In Italy, workers exposed to wood and leather dust undergo annual occupational-medicine screening to exclude dust-related sinonasal diseases, currently performed through anterior rhinoscopy, a procedure with significant diagnostic limitations. Since nasoendoscopy cannot be proposed for all workers due to cost and logistical constraints, nasal brushing could represent a practical alternative to improve diagnostic accuracy while reducing screening-management costs. If confirmed its reliability, it could be applied as a useful screening test in all workers exposed to wood and leather dust, who are frequently only screened by means of an anterior rhinoscopy. This could help reduce social and health care costs with a tool for early and minimally invasive diagnosis of sinonasal malignancies.

Overall, these findings support the robustness of our DNA methylation analysis and indicate that distinct epigenetic profiles characterize different sinonasal conditions. The complementary perspectives provided by PCA and UMAP strengthened the reliability of our classification approach, offering valuable insights for sinonasal tumours.

## Conclusions

The data presented suggest that the 13-DNA methylation method may serve as a valuable tool for the early diagnosis of sinonasal neoplastic lesions, enabling distinction between benign and malignant forms. Moreover, this method could also be beneficial for the medical monitoring of workers exposed to occupational sinonasal carcinogens. Further validation studies involving a larger number of cases are required to estimate this new assay’s diagnostic power.

## Supplementary Information

Supplementary Material 1. Methylation box plots and profile plots from all the 13 genes evaluated. For each group of samples, each line represents the methylation mean for each position. Asterisks indicate a statistical significance as calculated by the Kruskal-Wallis test. Summary Tables of each gene targets with the mean, the standard deviation, the minimum, the maximum, and the number of missing data for each position and group of samples. This test is the non-parametric version of the ANOVA (one-way analysis of variance) and tests whether samples originate from the same distribution. If the test is statistically significant (P value less than 0.05), it means that at least one of the samples is different from the other samples.

Supplementary Material 2. HeatMap considering all cases in which rows are centered; unit variance scaling is applied to rows. Both rows and columns are clustered using correlation distance and average linkage with 245 rows, 167 columns. Two clusters are marked: right cluster showed 54 normal samples, 19 inflammatory polyps, 6 tumours, 5 ITAC and 3 inverted papilloma; left cluster showed 20 tumours, 18 ITAC, 11 inverted papilloma, 14 inflammatory polyps and 17 normal cases.

Supplementary Material 3. Risk ratio (RR) and 95% CIs of obtaining a positive methylation score compared to normal mucosa.

## Data Availability

The original sequencing data presented in the study (FASTQ) are openly available in the NCBI Sequence Repository Archive (SRA) at PRJNA1322681.
