# Supplementary material for: DNA methylation analysis with nasal brushing for early diagnosis of sinonasal malignant tumours
Source: Discov Oncol. 2026 Jan 29;17:357. doi: 10.1007/s12672-026-04508-0 (PMC12923719; doi:10.1007/s12672-026-04508-0)
Supplement: Supplementary file 1 — Supplementary Material 1. Methylation box plots and profile plots from all the 13 genes evaluated. For each group of samples, each line represents the methylation mean for each position. Asterisks indicate a statistical significance as calculated by the Kruskal-Wallis test. Summary Tables of each gene targets with the mean, the standard deviation, the minimum, the maximum, and the number of missing data for each position and group of samples. This test is the non-parametric version of the ANOVA (one-way analysis of variance) and tests whether samples originate from the same distribution. If the test is statistically significant (P value less than 0.05), it means that at least one of the samples is different from the other samples. [file 12672_2026_4508_MOESM1_ESM.pdf]

EPHX3

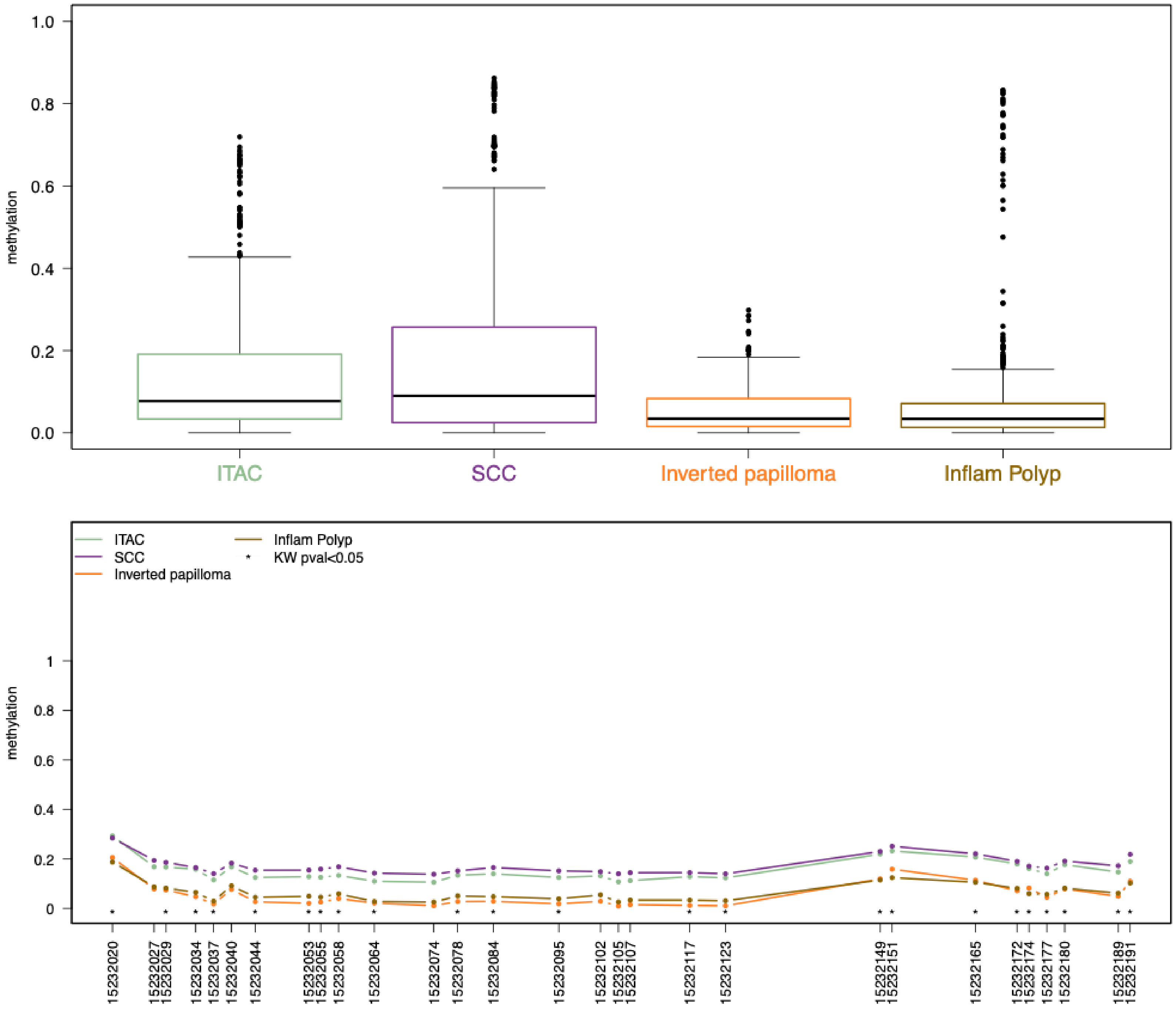

methylation
1.0
0.8
0.6
0.4
0.2
0.0
ITAC
SCC
Inverted papilloma
Inflam Polyp
ITAC
SCC
Inverted papilloma
Inflam Polyp
KW pval<0.05
methylation
1
0.8
0.6
0.4
0.2
0
15232020
15232027
15232029
15232034
15232037
15232040
15232044
15232053
15232055
15232058
15232064
15232074
15232078
15232084
15232095
15232102
15232105
15232107
15232117
15232123
15232149
15232151
15232165
15232172
15232174
15232177
15232180
15232189
15232191

EPHX3

	Position	ITAC Methyla	Inflam Polyp I	Inverted papi	SCC Methyla	ITAC Methyla	Inflam Polyp I	Inverted papi	SCC Methyla	ITAC Methyla	Inflam Polyp I	Inverted papi	SCC Methyla	ITAC Methyla	Inflam Polyp I	Inverted papi	SCC Methyla	ITAC Number	Inflam Polyp I	Inverted papi	SCC Number	Kruskall-Wal
V2	15232020	0.293219	0.1876472	0.2051583	0.2844552	0.16406555	0.13031249	0.06848706	0.19727342	0.0825	0.0268	0.0606	0.0176	0.7193	0.8242	0.2982	0.8289	0	0	0	0	2.368e-02
V3	15232027	0.1683619	0.08748611	0.078725	0.19394483	0.17799841	0.12836496	0.03378911	0.20977432	0.0155	0.0006	0	0	0.6764	0.8	0.1401	0.8492	0	0	0	0	5.166e-02
V4	15232029	0.1672571	0.082275	0.074225	0.1861034	0.17638395	0.13295675	0.03490853	0.1981101	0.0318	0.0016	0	0.0037	0.6656	0.8329	0.1231	0.797	0	0	0	0	8.896e-03
V5	15232034	0.15817143	0.06491389	0.047575	0.16521379	0.18328177	0.10816944	0.03111069	0.20969606	0.0181	0.0006	0	0	0.6636	0.6692	0.1077	0.8508	0	0	0	0	3.318e-02
V6	15232037	0.11530952	0.02953056	0.01814167	0.14078276	0.1793761	0.07743697	0.01182213	0.21121428	0	0	0	0	0.5804	0.4755	0.0352	0.8418	0	0	0	0	4.799e-02
V7	15232040	0.1682381	0.09146944	0.077025	0.1828	0.1795237	0.1257469	0.0364971	0.2085531	0.025	0.0024	0	0.002	0.6827	0.8042	0.147	0.819	0	0	0	0	3.063e-01
V8	15232044	0.12497619	0.04525833	0.02693333	0.15446552	0.16443945	0.10170093	0.01767815	0.21333667	0.0127	0.0018	0	0	0.6107	0.6141	0.0643	0.8297	0	0	0	0	5.618e-03
V9	15232053	0.1282619	0.049125	0.02088333	0.15482069	0.18733766	0.11655023	0.01485847	0.21582379	0	0.0022	0	0	0.6595	0.7182	0.0556	0.8379	0	0	0	0	2.564e-02
V10	15232055	0.12577143	0.04584722	0.02535833	0.15872759	0.16866332	0.11165781	0.01397097	0.2114051	0.014	0	0	0	0.5477	0.6887	0.0541	0.8189	0	0	0	0	2.879e-03
V11	15232058	0.13323333	0.059175	0.04013333	0.16848966	0.1713668	0.1190846	0.0248478	0.2077747	0	0.0017	0	0	0.6611	0.7413	0.0841	0.824	0	0	0	0	1.045e-02
V12	15232064	0.1096381	0.02809722	0.02131667	0.14281034	0.16897153	0.09894085	0.01272034	0.20303303	0	0	0.0048	0	0.6214	0.6007	0.0431	0.7816	0	0	0	0	1.040e-03
V13	15232074	0.10592381	0.02568333	0.01106667	0.13816207	0.18566846	0.09897695	0.01403355	0.21223519	0	0	0	0	0.6049	0.6007	0.0452	0.8275	0	0	0	0	1.065e-01
V14	15232078	0.13413333	0.05051944	0.02776667	0.15184138	0.1723792	0.1350616	0.0212903	0.2039221	0.0127	0	0	0	0.5804	0.8261	0.084	0.8393	0	0	0	0	1.804e-03
V15	15232084	0.14000476	0.04784444	0.02865	0.16543103	0.18248066	0.13132339	0.01762047	0.20465871	0	0	0	0	0.6544	0.8081	0.0736	0.8098	0	0	0	0	6.873e-05
V16	15232095	0.12501429	0.03916389	0.01864167	0.15176207	0.18042955	0.12655272	0.01344067	0.20814519	0.0018	0	0	0	0.6244	0.7721	0.0375	0.789	0	0	0	0	2.367e-03
V17	15232102	0.13140952	0.05475833	0.02896667	0.14866897	0.1843733	0.1314499	0.0194628	0.2041802	0	0	0	0	0.6244	0.812	0.0537	0.8447	0	0	0	0	9.760e-02
V18	15232105	0.10759048	0.02604722	0.009475	0.14015517	0.17405997	0.08996701	0.00518584	0.21717975	0	0	0	0	0.513	0.5437	0.0177	0.8189	0	0	0	0	5.224e-02
V19	15232107	0.11215238	0.03378333	0.01545833	0.14464483	0.17528788	0.11133734	0.01207043	0.21922549	0	0	0	0	0.5313	0.6779	0.0327	0.8455	0	0	0	0	1.856e-01
V20	15232117	0.12840952	0.03363333	0.01223333	0.14497586	0.1838687	0.12710994	0.00881686	0.21743506	0.0018	0	0	0	0.6321	0.772	0.0276	0.8423	0	0	0	0	1.303e-03
V21	15232123	0.12335714	0.03101667	0.01051667	0.1401	0.18347043	0.10361709	0.0077096	0.21196148	0	0	0	0	0.6376	0.6286	0.0276	0.8239	0	0	0	0	3.268e-03
V22	15232149	0.2192857	0.114675	0.1195833	0.2301	0.16351185	0.13372904	0.05506162	0.18963751	0.0348	0.0059	0	0.0215	0.6672	0.8309	0.1775	0.8383	0	0	0	0	2.768e-04
V23	15232151	0.2322857	0.1244111	0.1586833	0.2514172	0.15578757	0.11779019	0.04908614	0.19125661	0.0435	0.0031	0.0921	0.0235	0.6947	0.7188	0.2461	0.8381	0	0	0	0	2.396e-04
V24	15232165	0.2075619	0.1056306	0.1139417	0.2205414	0.17325089	0.11681794	0.05921106	0.19540609	0.0348	0.0036	0	0.0207	0.6748	0.7246	0.2015	0.8373	0	0	0	0	2.500e-03
V25	15232172	0.17948571	0.08105278	0.07101667	0.19032759	0.18202661	0.11945836	0.04103612	0.20432741	0.0261	0	0	0.0007	0.6529	0.744	0.1321	0.8361	0	0	0	0	6.124e-03
V26	15232174	0.16118095	0.06001667	0.08258333	0.17075862	0.17776579	0.09369873	0.06466973	0.20706169	0.017	0.0006	0.0184	0	0.6733	0.5648	0.2727	0.8225	0	0	0	0	2.278e-03
V27	15232177	0.14016667	0.056975	0.04433333	0.16308966	0.18220665	0.12584658	0.02509293	0.21021868	0.0116	0	0	0	0.6489	0.7738	0.0708	0.8474	0	0	0	0	1.733e-02
V28	15232180	0.17690476	0.08191111	0.079075	0.19132069	0.17517354	0.12072718	0.03007981	0.20253314	0.0413	0.0034	0	0.0052	0.657	0.7477	0.1131	0.8258	0	0	0	0	3.800e-03
V29	15232189	0.14703333	0.06173056	0.048825	0.17213448	0.17987279	0.1067566	0.02380161	0.21435367	0	0	0	0	0.6336	0.6615	0.0871	0.8623	0	0	0	0	2.214e-02
V30	15232191	0.1895857	0.1020306	0.1101083	0.218131	0.17500272	0.1252762	0.04854925	0.2101652	0.0287	0.0105	0	0	0.687	0.7784	0.1907	0.854	0	0	0	0	7.519e-03

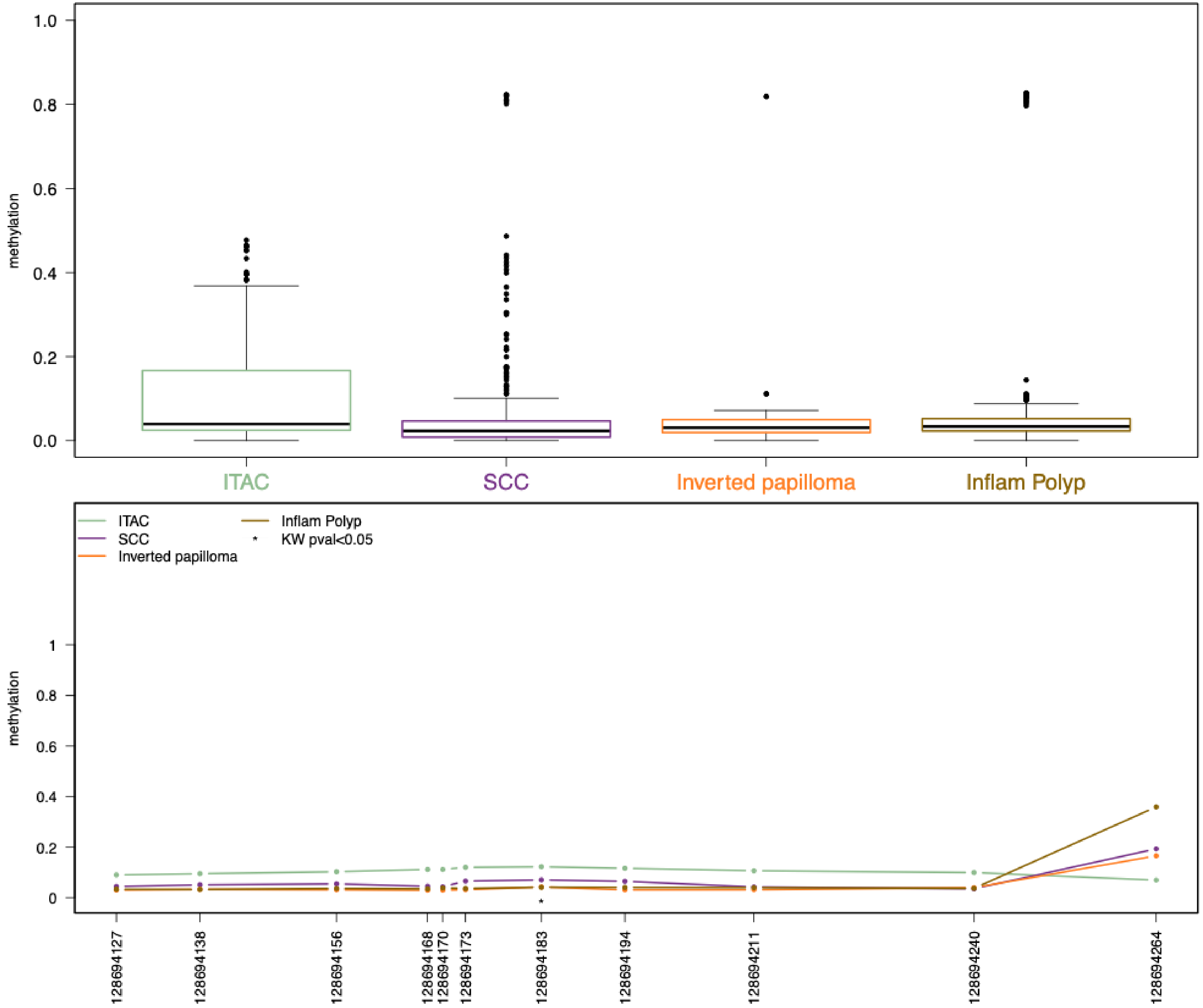

1.0
0.8
0.6
0.4
0.2
0.0
methylation
ITAC
SCC
Inverted papilloma
Inflam Polyp
ITAC
SCC
Inverted papilloma
Inflam Polyp
* KW pval<0.05
1
0.8
0.6
0.4
0.2
0
methylation
128694127
128694138
128694156
128694168
128694170
128694173
128694183
128694194
128694211
128694240
128694264

FLI1

FLI1

	Position	ITAC Methyla	Inflam Polyp I	Inverted papi	SCC Methyla	ITAC Methyla	Inflam Polyp I	Inverted papi	SCC Methyla	ITAC Methyla	Inflam Polyp I	Inverted papi	SCC Methyla	ITAC Methyla	Inflam Polyp I	Inverted papi	SCC Methyla	ITAC Number	Inflam Polyp I	Inverted papi	SCC Number	Kruskall-Wal
V2	128694127	0.09028571	0.03348333	0.03020833	0.04459655	0.11979953	0.02223789	0.01968938	0.07056707	0	0	0	0	0.4335	0.0984	0.0581	0.349	0	0	0	0	0.24433
V3	128694138	0.09512381	0.03271667	0.032825	0.05087931	0.11987517	0.01978378	0.01681277	0.08289256	0	0	0	0	0.4012	0.0749	0.0563	0.3986	0	0	0	0	0.52132
V4	128694156	0.1029381	0.03668889	0.03131667	0.0545931	0.12557143	0.02313875	0.02165607	0.07726755	0.0032	0	0	0	0.4522	0.1036	0.0607	0.305	0	0	0	0	0.54715
V5	128694168	0.11154286	0.03506667	0.029475	0.04533103	0.13902381	0.02226646	0.01959755	0.070399	0	0	0.0024	0	0.477	0.0976	0.0631	0.3001	0	0	0	0	0.10067
V6	128694170	0.11155238	0.03872222	0.02945833	0.04326552	0.13576741	0.02430102	0.01952506	0.06809531	0.0087	0	0	0	0.4523	0.1079	0.0658	0.3352	0	0	0	0	0.08627
V7	128694173	0.12010952	0.03646389	0.031975	0.06586897	0.13417918	0.02390843	0.02089942	0.11645089	0.0067	0	0.0063	0	0.4653	0.107	0.0666	0.4412	0	0	0	0	0.07355
V8	128694183	0.12255238	0.04153611	0.04296667	0.07046207	0.13555986	0.02844631	0.02769632	0.1275838	0.0079	0	0.0077	0	0.46	0.1441	0.1114	0.4865	0	0	0	0	0.03887
V9	128694194	0.11621905	0.04072778	0.031275	0.0649	0.13709235	0.0259906	0.02263951	0.11438453	0.0067	0	0.0024	0	0.4635	0.1103	0.0695	0.4344	0	0	0	0	0.09545
V10	128694211	0.10666667	0.03989722	0.03168333	0.04269655	0.12922647	0.02425788	0.02186666	0.06140827	0	0	0	0	0.4624	0.1089	0.0681	0.2521	0	0	0	0	0.06645
V11	128694240	0.09965714	0.03713611	0.03978333	0.03553448	0.1127969	0.02375525	0.030546	0.03868365	0.0071	0	0	0	0.3958	0.098	0.1114	0.1632	0	0	0	0	0.13335
V12	128694264	0.06960476	0.35910278	0.16555833	0.1932	0.08586955	0.39228471	0.305683	0.32451752	0.0136	0	0	0	0.3681	0.8274	0.8188	0.8235	0	0	0	0	0.14186

GP1BB

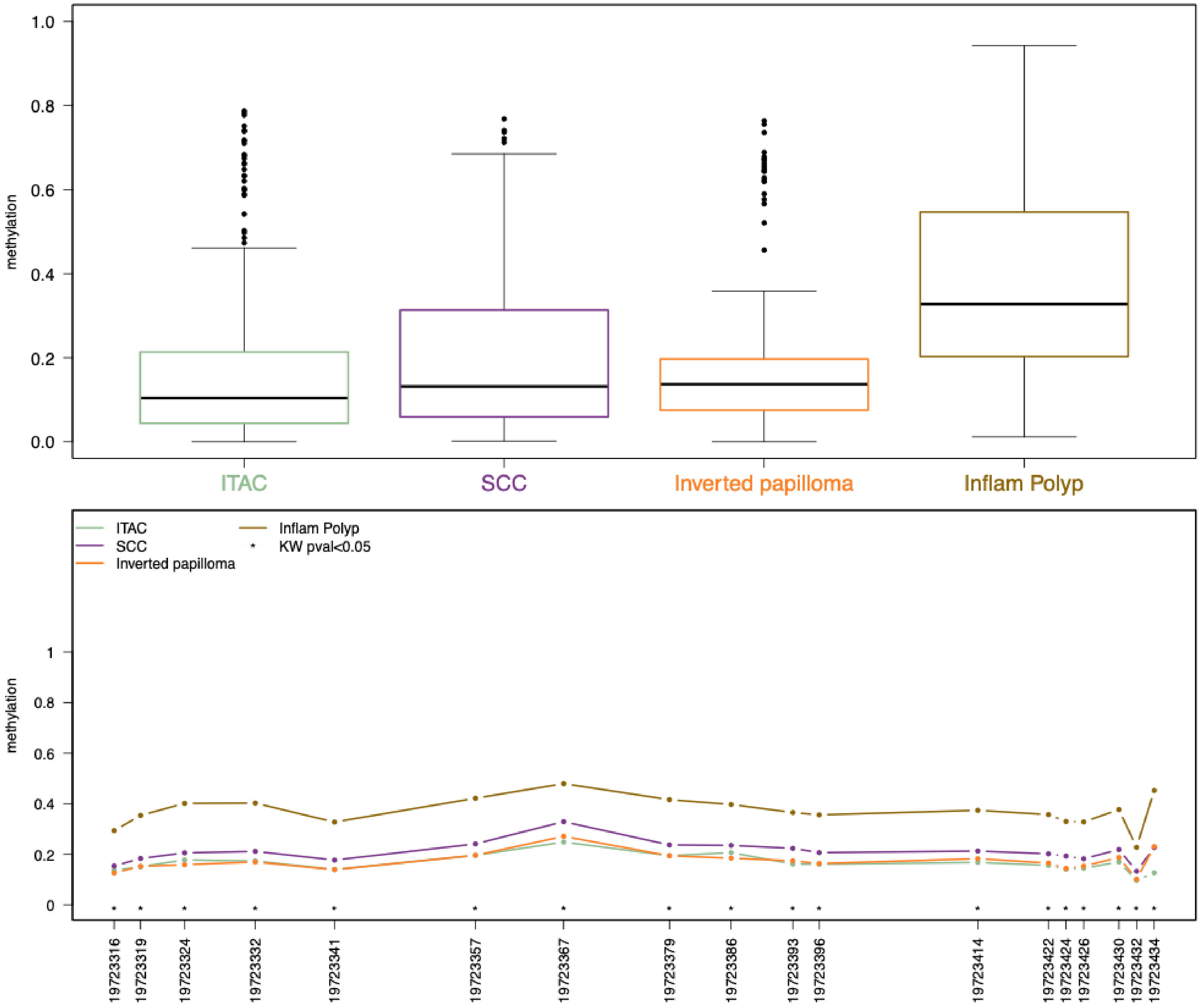

1.0
0.8
0.6
0.4
0.2
0.0
methylation
ITAC
SCC
Inverted papilloma
Inflam Polyp
ITAC
SCC
Inverted papilloma
Inflam Polyp
KW pval<0.05
1
0.8
0.6
0.4
0.2
0
methylation
19723316
19723319
19723324
19723332
19723341
19723357
19723367
19723379
19723386
19723393
19723396
19723414
19723422
19723424
19723426
19723430
19723432
19723434

GP1BB

	Position	ITAC Methyla	Inflam Polyp I	Inverted papi	SCC Methyla	ITAC Methyla	Inflam Polyp I	Inverted papi	SCC Methyla	ITAC Methyla	Inflam Polyp I	Inverted papi	SCC Methyla	ITAC Methyla	Inflam Polyp I	Inverted papi	SCC Methyla	ITAC Number	Inflam Polyp I	Inverted papi	SCC Number	Kruskall-Wal
V2	19723316	0.1368	0.2936917	0.1262083	0.1539552	0.1461516	0.1775088	0.1312925	0.1589337	0	0.0126	0.012	0.0096	0.4974	0.672	0.5211	0.5188	0	0	0	0	1.705e-04
V3	19723319	0.1511571	0.3538278	0.1525083	0.1834724	0.1978268	0.2163591	0.156264	0.1825082	0	0.0138	0.0128	0.0088	0.7876	0.7571	0.6193	0.6045	0	0	0	0	1.502e-04
V4	19723324	0.1777571	0.4014194	0.1588833	0.2057897	0.2122214	0.246882	0.1760522	0.2105861	0	0.031	0.0086	0.0104	0.7824	0.8745	0.6889	0.7124	0	0	0	0	1.914e-04
V5	19723332	0.1735762	0.4025333	0.16965	0.2116793	0.1925583	0.237248	0.1686108	0.2091496	0	0.0289	0	0.0241	0.6836	0.8668	0.6633	0.6841	0	0	0	0	1.408e-04
V6	19723341	0.1402429	0.3275361	0.1392417	0.1771621	0.1522128	0.2018164	0.1636779	0.1746291	0	0.0115	0	0.0017	0.59	0.7982	0.6229	0.601	0	0	0	0	1.989e-04
V7	19723357	0.1960667	0.4217583	0.1961583	0.2413138	0.2115572	0.237456	0.1883515	0.2126859	0	0.0674	0.03	0.0233	0.7513	0.8617	0.7556	0.7412	0	0	0	0	1.528e-04
V8	19723367	0.2486571	0.4794167	0.2711917	0.3290483	0.1578292	0.2154002	0.1747969	0.1930144	0.0044	0.1332	0.0563	0.0835	0.7181	0.9087	0.7638	0.768	0	0	0	0	9.557e-05
V9	19723379	0.1945524	0.4160944	0.194125	0.2371931	0.2074067	0.2450315	0.1844369	0.2193305	0	0.0505	0.0171	0.0217	0.7409	0.9422	0.7361	0.7367	0	0	0	0	2.660e-04
V10	19723386	0.2066333	0.3970278	0.1846583	0.2353276	0.2335096	0.2330368	0.1678637	0.2106557	0	0.0401	0.03	0.0281	0.7772	0.8981	0.6786	0.6667	0	0	0	0	6.624e-04
V11	19723393	0.1616238	0.3652667	0.1741833	0.2235	0.1444264	0.206279	0.1610935	0.1867042	0.0345	0.0587	0.0248	0.0217	0.6479	0.7705	0.651	0.6372	0	0	0	0	1.303e-04
V12	19723396	0.1606952	0.3560944	0.1635667	0.2067517	0.1792969	0.2092993	0.1560862	0.1891323	0	0.0126	0.0197	0.0225	0.6736	0.833	0.6281	0.6102	0	0	0	0	1.618e-04
V13	19723414	0.1681333	0.3739944	0.1826083	0.2129966	0.1557363	0.2165226	0.1690252	0.1892572	0.0099	0.0512	0.0223	0.0337	0.6337	0.8497	0.6704	0.6162	0	0	0	0	1.717e-04
V14	19723422	0.1560333	0.3572083	0.1653333	0.2020103	0.1729655	0.2242545	0.1650968	0.2008541	0	0.0321	0.0172	0.0218	0.6607	0.8411	0.6569	0.6309	0	0	0	0	1.718e-04
V15	19723424	0.1409571	0.3298972	0.1442667	0.1930379	0.1738603	0.1921266	0.1551261	0.1981544	0	0.0347	0	0.0137	0.601	0.6998	0.59	0.6343	0	0	0	0	1.190e-04
V16	19723426	0.1446952	0.3281556	0.1533917	0.1822759	0.1479613	0.1988803	0.1461785	0.1632146	0.0297	0.0149	0.0172	0.0177	0.6205	0.7586	0.5769	0.5074	0	0	0	0	2.107e-04
V17	19723430	0.1698905	0.3769361	0.187175	0.2188621	0.1559529	0.2102218	0.1657094	0.1912377	0	0.0438	0.0301	0.0337	0.6025	0.7921	0.6733	0.6343	0	0	0	0	1.558e-04
V18	19723432	0.0969619	0.2275278	0.1014083	0.133231	0.1106571	0.1405872	0.118528	0.1390625	0.0044	0.0274	0.0129	0.0064	0.3698	0.524	0.4564	0.5	0	0	0	0	2.689e-04
V19	19723434	0.1268143	0.4522917	0.2305917	0.2265655	0.1485719	0.219955	0.2386738	0.238867	0	0.0408	0.0164	0.0121	0.5993	0.8713	0.6441	0.6853	0	0	0	0	6.929e-06

ITGA4

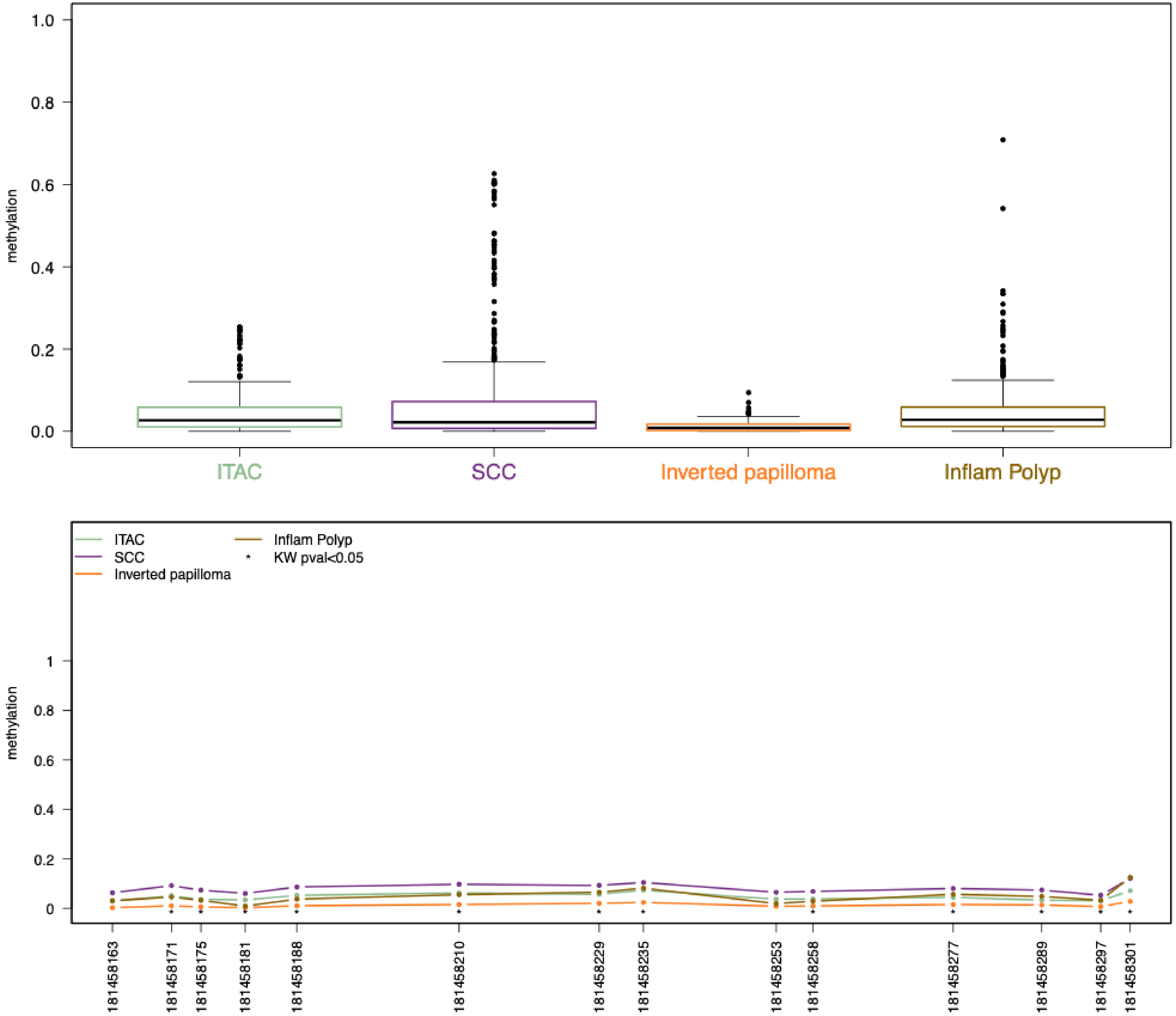

methylation
1.0
0.8
0.6
0.4
0.2
0.0
ITAC
SCC
Inverted papilloma
Inflam Polyp
ITAC
SCC
Inverted papilloma
Inflam Polyp
KW pval<0.05
methylation
1
0.8
0.6
0.4
0.2
0
181458163
181458171
181458175
181458181
181458188
181458210
181458229
181458235
181458253
181458258
181458277
181458289
181458297
181458301

ITGA4

	Position	ITAC Methyla	Inflam Polyp I	Inverted papi	SCC Methyla	ITAC Methyla	Inflam Polyp I	Inverted papi	SCC Methyla	ITAC Methyla	Inflam Polyp I	Inverted papi	SCC Methyla	ITAC Methyla	Inflam Polyp I	Inverted papi	SCC Methyla	ITAC Number	Inflam Polyp I	Inverted papi	SCC Number	Kruskall-Wal
V2	181458163	0.02990952	0.03077222	0.002875	0.06358621	0.0565208	0.11778134	0.00293788	0.14143517	0	0	0	0	0.2214	0.7086	0.0086	0.5841	0	0	0	0	0.0845008
V3	181458171	0.05123333	0.04591944	0.01035833	0.09193448	0.06728745	0.05654579	0.00805249	0.16550257	0	0.0013	0	0.0017	0.2462	0.3343	0.0234	0.6062	0	0	0	0	0.0043461
V4	181458175	0.03602381	0.03265833	0.00606667	0.07377931	0.05931901	0.05668332	0.00631813	0.14233457	0	0	0	0	0.2456	0.3343	0.0223	0.5691	0	0	0	0	0.0302979
V5	181458181	0.03434762	0.00917222	0.00270833	0.06048966	0.05606477	0.01669875	0.00392207	0.13613036	0	0	0	0	0.212	0.0964	0.0137	0.602	0	0	0	0	0.0245883
V6	181458188	0.05264286	0.03771944	0.01069167	0.08672759	0.06916335	0.05269221	0.00776431	0.16282599	0	0	0	0	0.2535	0.3092	0.0257	0.6002	0	0	0	0	0.0307041
V7	181458210	0.0624381	0.05564722	0.01565	0.09743448	0.06537569	0.05457101	0.0131043	0.15239279	0	0	0.0021	0.0011	0.2494	0.2869	0.0451	0.6057	0	0	0	0	0.0101048
V8	181458229	0.05697619	0.06541111	0.020475	0.09288621	0.056792	0.0502653	0.01827965	0.14637863	0	0.0057	0.0004	0.0007	0.2207	0.2568	0.0571	0.6034	0	0	0	0	0.0095058
V9	181458235	0.07332857	0.08207222	0.02429167	0.10493448	0.06581707	0.05237855	0.02146686	0.14933372	0.0067	0.004	0	0	0.2542	0.2451	0.0701	0.6104	0	0	0	0	0.0031055
V10	181458253	0.03725714	0.01962222	0.00833333	0.06524138	0.05130194	0.02074311	0.00725802	0.14485252	0	0	0	0	0.2028	0.0885	0.0183	0.6264	0	0	0	0	0.2270532
V11	181458258	0.03815238	0.02906389	0.00956667	0.0688	0.04835195	0.02988253	0.00842306	0.13400635	0.0016	0	0	0	0.2227	0.156	0.0215	0.5764	0	0	0	0	0.0446924
V12	181458277	0.04440476	0.05704444	0.015775	0.08079655	0.04889795	0.04939451	0.01228437	0.13910065	0.0017	0.002	0.0007	0.0009	0.2227	0.2903	0.042	0.6021	0	0	0	0	0.0019071
V13	181458289	0.03371429	0.0486	0.013975	0.07421724	0.03847349	0.05782298	0.01045876	0.11707377	0	0	0	0	0.1748	0.3419	0.0343	0.4388	0	0	0	0	0.0198028
V14	181458297	0.03098095	0.03279722	0.00714167	0.05294828	0.03964803	0.04282531	0.00720145	0.07943157	9e-04	0e+00	0e+00	0e+00	0.1776	0.2497	0.0245	0.3156	0	0	0	0	0.0086025
V15	181458301	0.07138095	0.12584722	0.02816667	0.12107241	0.06771213	0.09654649	0.02583814	0.15337259	0	0.0044	0	0.0007	0.2301	0.5416	0.094	0.5827	0	0	0	0	0.0003192

KIF1A

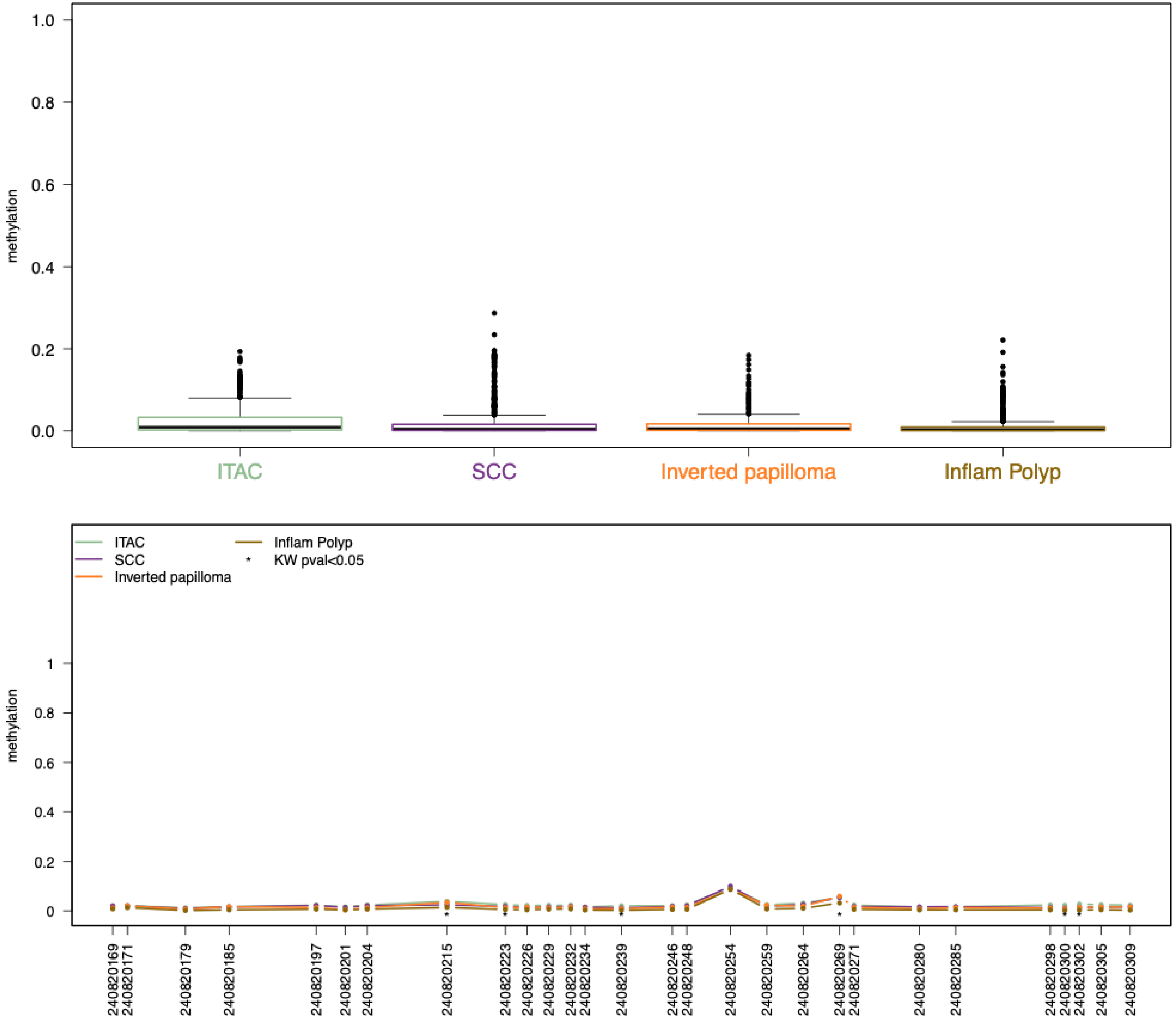

methylation
1.0
0.8
0.6
0.4
0.2
0.0
ITAC
SCC
Inverted papilloma
Inflam Polyp
ITAC
SCC
Inverted papilloma
Inflam Polyp
KW pval<0.05
methylation
1
0.8
0.6
0.4
0.2
0
240820169
240820171
240820179
240820185
240820197
240820201
240820204
240820215
240820223
240820226
240820229
240820232
240820234
240820239
240820246
240820248
240820254
240820259
240820264
240820269
240820271
240820280
240820285
240820298
240820300
240820302
240820305
240820309

KIF1A

	Position	ITAC Methyla	Inflam Polyp	Inverted papi	SCC Methyla	ITAC Methyla	Inflam Polyp	Inverted papi	SCC Methyla	ITAC Methyla	Inflam Polyp	Inverted papi	SCC Methyla	ITAC Methyla	Inflam Polyp	Inverted papi	SCC Methyla	ITAC Number	Inflam Polyp	Inverted papi	SCC Number	Kruskall-Wal
V2	240820169	0.02085238	0.00817778	0.01228333	0.02005517	0.02944584	0.00824777	0.02121667	0.03866628	0e+00	0e+00	0e+00	6e-04	0.1347	0.04	0.074	0.1813	0	0	0	0	0.151444
V3	240820171	0.021	0.01304444	0.02095833	0.02046552	0.02963874	0.03639075	0.04547464	0.04091029	0	0	0	0	0.1285	0.2216	0.1621	0.1793	0	0	0	0	0.44827
V4	240820179	0.01265714	0.00161389	0.00729167	0.00985862	0.02767507	0.00319089	0.01161202	0.03371466	0	0	0	0	0.1188	0.0139	0.0417	0.1742	0	0	0	0	0.127702
V5	240820185	0.0177	0.00423056	0.017225	0.01563103	0.03236925	0.00806962	0.02933754	0.0367018	0	0	0	0	0.1273	0.0409	0.0817	0.1802	0	0	0	0	0.248923
V6	240820197	0.02348095	0.0069	0.01365	0.02224138	0.03401902	0.00805488	0.02625735	0.04727518	0	0	0	0	0.1304	0.0297	0.091	0.1849	0	0	0	0	0.129581
V7	240820201	0.01660476	0.00395	0.004725	0.01417586	0.03163884	0.00542342	0.00902281	0.03566572	0	0	0	0	0.1276	0.0207	0.0322	0.183	0	0	0	0	0.372713
V8	240820204	0.02375238	0.00703611	0.01293333	0.02134483	0.03658837	0.00906075	0.01985732	0.04562249	0	0	0	0	0.1335	0.0433	0.0689	0.1848	0	0	0	0	0.14463
V9	240820215	0.03881905	0.01409167	0.03473333	0.02477931	0.03703884	0.01188392	0.04891853	0.04467065	0.0037	0	0.0003	0.0015	0.1356	0.0531	0.174	0.1868	0	0	0	0	0.007576
V10	240820223	0.02550476	0.00549167	0.01303333	0.0180069	0.03490799	0.00704226	0.0230556	0.03573708	0	0	0	0	0.1245	0.0256	0.081	0.1766	0	0	0	0	0.023009
V11	240820226	0.02211429	0.00441389	0.01544167	0.01391724	0.03212286	0.00572828	0.02742761	0.02528303	0	0	0	0	0.1247	0.024	0.0944	0.1224	0	0	0	0	0.058475
V12	240820229	0.02266667	0.00680833	0.01265833	0.01614828	0.03226342	0.00907075	0.01622655	0.0346951	0	0	0	0	0.1284	0.0385	0.0539	0.1753	0	0	0	0	0.171684
V13	240820232	0.02340476	0.00791944	0.016075	0.01814828	0.0325903	0.00969406	0.03085512	0.03785256	0	0	0	0	0.1271	0.0433	0.1106	0.1853	0	0	0	0	0.293002
V14	240820234	0.01594762	0.00353333	0.00833333	0.01513448	0.02814951	0.00528691	0.01307366	0.03860994	0	0	0	0	0.1246	0.0214	0.0468	0.1771	0	0	0	0	0.183383
V15	240820239	0.02067143	0.00247778	0.00934167	0.01185517	0.03114439	0.00490709	0.01790899	0.03452454	0	0	0	0	0.1219	0.0225	0.0609	0.1776	0	0	0	0	0.022401
V16	240820246	0.02104286	0.00596389	0.01476667	0.01797241	0.03367892	0.00633337	0.02928752	0.03601987	0	0	0	0	0.1307	0.0272	0.1019	0.17	0	0	0	0	0.439336
V17	240820248	0.02433333	0.00638333	0.014425	0.02017586	0.03385728	0.00616935	0.02540888	0.03765647	0	0	0	0	0.1351	0.0227	0.0879	0.1857	0	0	0	0	0.115232
V18	240820254	0.09441429	0.08713056	0.08709167	0.09942759	0.03744759	0.03397623	0.02383212	0.03382797	0.05	0.0236	0.0423	0.032	0.1934	0.1915	0.1187	0.196	0	0	0	0	0.449663
V19	240820259	0.02510952	0.00773056	0.02280833	0.01819655	0.03316504	0.00799442	0.02789091	0.0279114	0	0	0.0017	0	0.1317	0.0305	0.0939	0.1373	0	0	0	0	0.065496
V20	240820264	0.03135714	0.01038056	0.01939167	0.02601724	0.03875025	0.01137377	0.0353366	0.0588618	0.0016	0	0	0	0.1298	0.0493	0.1281	0.2869	0	0	0	0	0.119406
V21	240820269	0.05494286	0.03217222	0.05945	0.05383448	0.03967184	0.02225653	0.05549361	0.04889123	0.016	0.0005	0	0.0125	0.1672	0.0997	0.1842	0.2344	0	0	0	0	0.044041
V22	240820271	0.02381905	0.00626944	0.01479167	0.01778966	0.03102896	0.00657809	0.03207365	0.04062049	4e-04	0e+00	0e+00	0e+00	0.1279	0.0279	0.1155	0.1739	0	0	0	0	0.086215
V23	240820280	0.01537143	0.003925	0.00845	0.01652069	0.0278522	0.00582906	0.01716362	0.03919488	0	0	0	0	0.122	0.0288	0.0577	0.1593	0	0	0	0	0.389211
V24	240820285	0.01639524	0.0043	0.01326667	0.01727241	0.02912562	0.00707402	0.02472027	0.03774649	0	0	0	0	0.1221	0.0364	0.0891	0.1575	0	0	0	0	0.152786
V25	240820298	0.0242381	0.00411944	0.01088333	0.01313103	0.04725173	0.00532309	0.01937552	0.0336458	0	0	0	0	0.1783	0.0246	0.0702	0.1654	0	0	0	0	0.256044
V26	240820300	0.02279048	0.00185833	0.00836667	0.01073793	0.04575567	0.00341261	0.01537663	0.03228993	0	0	0	0	0.1732	0.0198	0.0541	0.167	0	0	0	0	0.04869
V27	240820302	0.02755238	0.00331667	0.01424167	0.01467241	0.0475655	0.00533482	0.02405189	0.03169229	0	0	0	0	0.1717	0.0246	0.0757	0.1609	0	0	0	0	0.010295
V28	240820305	0.0252	0.00455278	0.01560833	0.01523793	0.04099722	0.00628643	0.02080347	0.03180714	0e+00	0e+00	6e-04	0e+00	0.132	0.0256	0.0679	0.144	0	0	0	0	0.076375
V29	240820309	0.02355238	0.00312778	0.01608333	0.01655172	0.04243899	0.00476233	0.03790792	0.03859158	0	0	0	0	0.1261	0.0268	0.1345	0.1568	0	0	0	0	0.075883

LINC0599

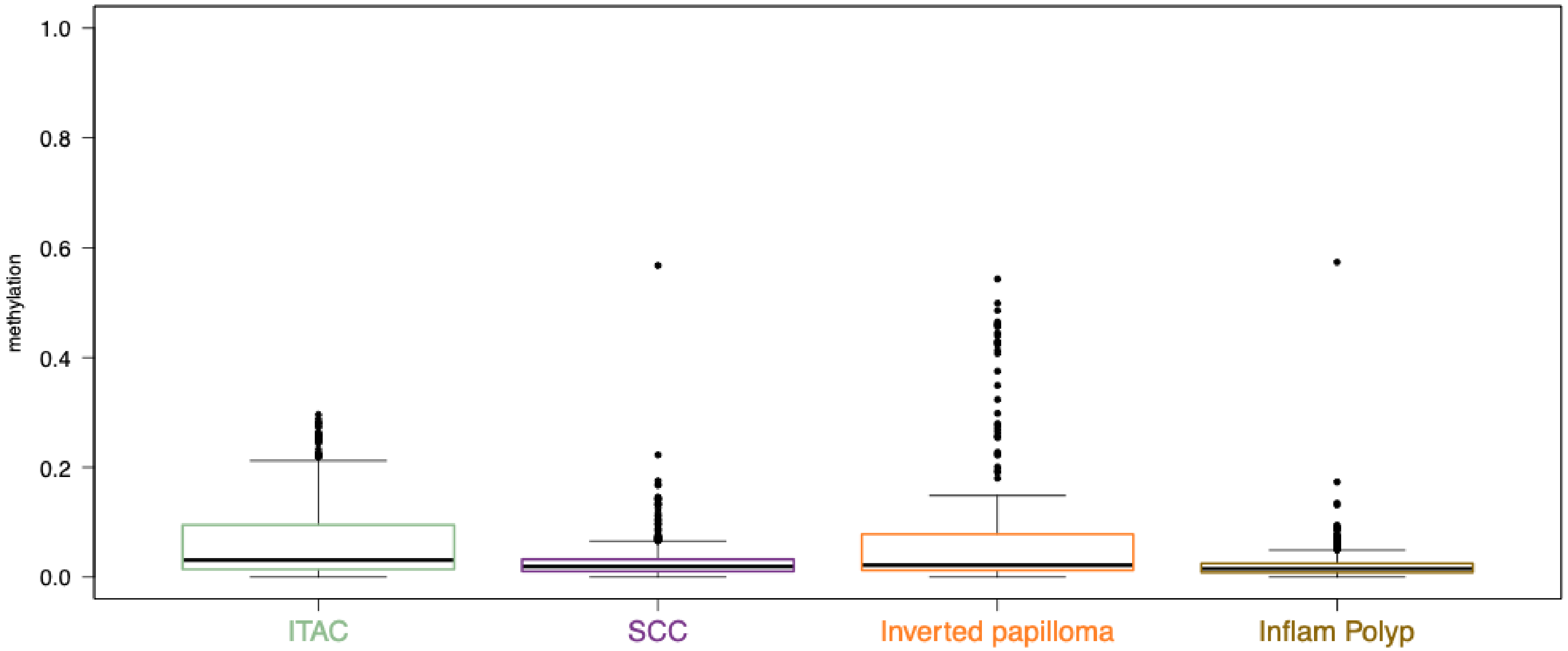

1.0
0.8
0.6
0.4
0.2
0.0
methylation
ITAC
SCC
Inverted papilloma
Inflam Polyp

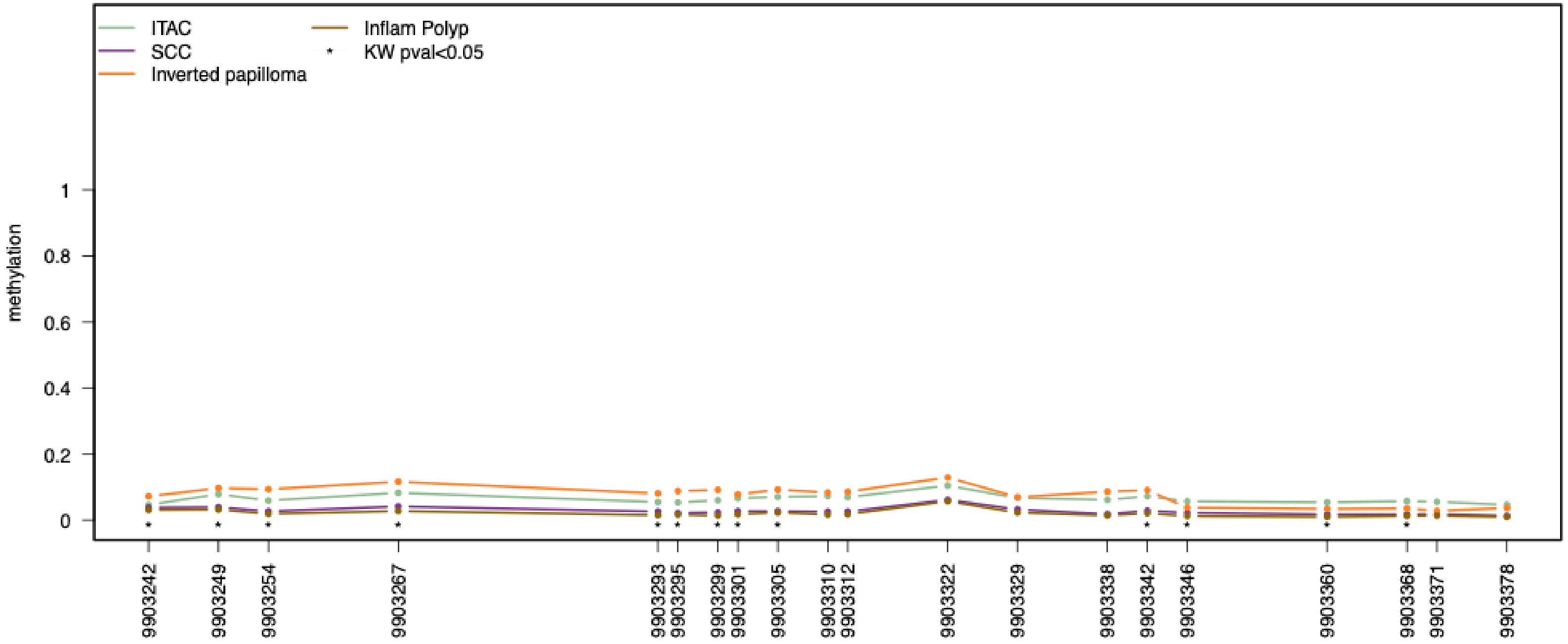

ITAC
SCC
Inverted papilloma
Inflam Polyp
* KW pval<0.05
1
0.8
0.6
0.4
0.2
0
methylation
9903242
9903249
9903254
9903267
9903293
9903295
9903299
9903301
9903305
9903310
9903312
9903322
9903329
9903338
9903342
9903346
9903360
9903368
9903371
9903378

LINC0599

	Position	ITAC Methyla	Inflam Polyp I	Inverted papi	SCC Methyla	ITAC Methyla	Inflam Polyp I	Inverted papi	SCC Methyla	ITAC Methyla	Inflam Polyp I	Inverted papi	SCC Methyla	ITAC Methyla	Inflam Polyp I	Inverted papi	SCC Methyla	ITAC Number	Inflam Polyp I	Inverted papi	SCC Number	Kruskall-Wal
V2	9903242	0.04659048	0.03138333	0.07273333	0.0381931	0.0556606	0.09366035	0.12539434	0.10351335	0.0083	0	0.0088	0.0002	0.2484	0.5738	0.429	0.5676	0	0	0	0	0.029034
V3	9903249	0.07911429	0.03234444	0.09731667	0.03954828	0.07518323	0.01963837	0.12963998	0.03026679	0.0142	0.0008	0.0127	0.0031	0.2798	0.0928	0.462	0.1688	0	0	0	0	0.019254
V4	9903254	0.05955238	0.01920833	0.09389167	0.02706552	0.06679545	0.01246218	0.15993476	0.02977694	0.01	0	0.0092	0.0002	0.2559	0.0626	0.4399	0.1453	0	0	0	0	0.039535
V5	9903267	0.08317143	0.02775556	0.116675	0.04191724	0.07188226	0.01708788	0.17332913	0.03760491	0.0191	0	0.0166	0.0055	0.2748	0.0942	0.4987	0.1753	0	0	0	0	0.00109
V6	9903293	0.05488095	0.01526667	0.08143333	0.0263069	0.06899299	0.01222716	0.1312083	0.02181025	0.0006	0.0012	0	0	0.2818	0.0501	0.4236	0.0718	0	0	0	0	0.001702
V7	9903295	0.05360476	0.01693056	0.088525	0.02191724	0.06218298	0.0119163	0.13894624	0.0174969	0.0006	0	0.0094	0	0.2514	0.0586	0.4588	0.0717	0	0	0	0	0.002915
V8	9903299	0.06049048	0.01405	0.09190833	0.02371034	0.07928597	0.01215325	0.15185758	0.02079235	0.0031	0	0	0	0.2591	0.0452	0.4449	0.0842	0	0	0	0	0.036273
V9	9903301	0.06640952	0.01793056	0.078575	0.02738621	0.07650365	0.010922	0.1243612	0.02020654	0.0074	0	0.0087	0	0.296	0.0458	0.4073	0.0969	0	0	0	0	0.014858
V10	9903305	0.07085714	0.02320556	0.09278333	0.02725862	0.08031864	0.0192593	0.1359663	0.02497794	0.0047	0.0008	0.0058	0	0.2569	0.0843	0.4566	0.1051	0	0	0	0	0.034591
V11	9903310	0.0729381	0.01698611	0.08416667	0.026	0.0871797	0.01359181	0.13312476	0.02872358	0.0059	0	0.0043	0	0.2601	0.0699	0.4132	0.1423	0	0	0	0	0.085122
V12	9903312	0.06990476	0.01819167	0.08636667	0.02613448	0.08771546	0.01395852	0.12951393	0.02759578	0	0	0.0082	0	0.2583	0.0649	0.4282	0.1431	0	0	0	0	0.083746
V13	9903322	0.10484762	0.05761944	0.1296	0.06178276	0.08841362	0.03536926	0.15241155	0.0481924	0.0166	0	0.0188	0.0126	0.2877	0.1733	0.5431	0.2225	0	0	0	0	0.197021
V14	9903329	0.06747619	0.02303889	0.070125	0.03270345	0.07638802	0.01325537	0.10305914	0.02985068	0.0011	0.005	0.0029	0.0035	0.2492	0.0708	0.3491	0.133	0	0	0	0	0.190346
V15	9903338	0.06168095	0.01441111	0.08689167	0.01830345	0.07773998	0.01474323	0.15522886	0.02554631	0.0012	0	0	0	0.2471	0.0633	0.4857	0.1326	0	0	0	0	0.107318
V16	9903342	0.07258571	0.02093611	0.09105833	0.02881034	0.08401596	0.02032402	0.13870368	0.03075226	9e-03	0e+00	8e-03	4e-04	0.2643	0.0876	0.4595	0.1416	0	0	0	0	0.039105
V17	9903346	0.05717143	0.01261667	0.03848333	0.02250345	0.07924048	0.00941016	0.06174482	0.02441913	6e-04	0e+00	0e+00	0e+00	0.26	0.0408	0.2245	0.1327	0	0	0	0	0.035022
V18	9903360	0.05414286	0.01025	0.034425	0.01745517	0.07092325	0.009371	0.05224133	0.02425532	0.0043	0	0	0	0.2516	0.0363	0.1909	0.1319	0	0	0	0	0.009218
V19	9903368	0.05777619	0.01309167	0.03584167	0.01736207	0.06651514	0.00853217	0.0444829	0.02024504	0.0027	0	0.0082	0.0006	0.2199	0.0306	0.1391	0.111	0	0	0	0	0.02043
V20	9903371	0.05572857	0.01375	0.028575	0.01836207	0.06765553	0.00895961	0.0375469	0.02372793	0.0046	0	0.0058	0	0.2253	0.0387	0.1407	0.1259	0	0	0	0	0.081111
V21	9903378	0.0469619	0.01000833	0.0373	0.01321379	0.05773012	0.0073095	0.06094724	0.02151761	0.0011	0	0.001	0	0.1915	0.0344	0.1941	0.1118	0	0	0	0	0.097082

LRRTM1

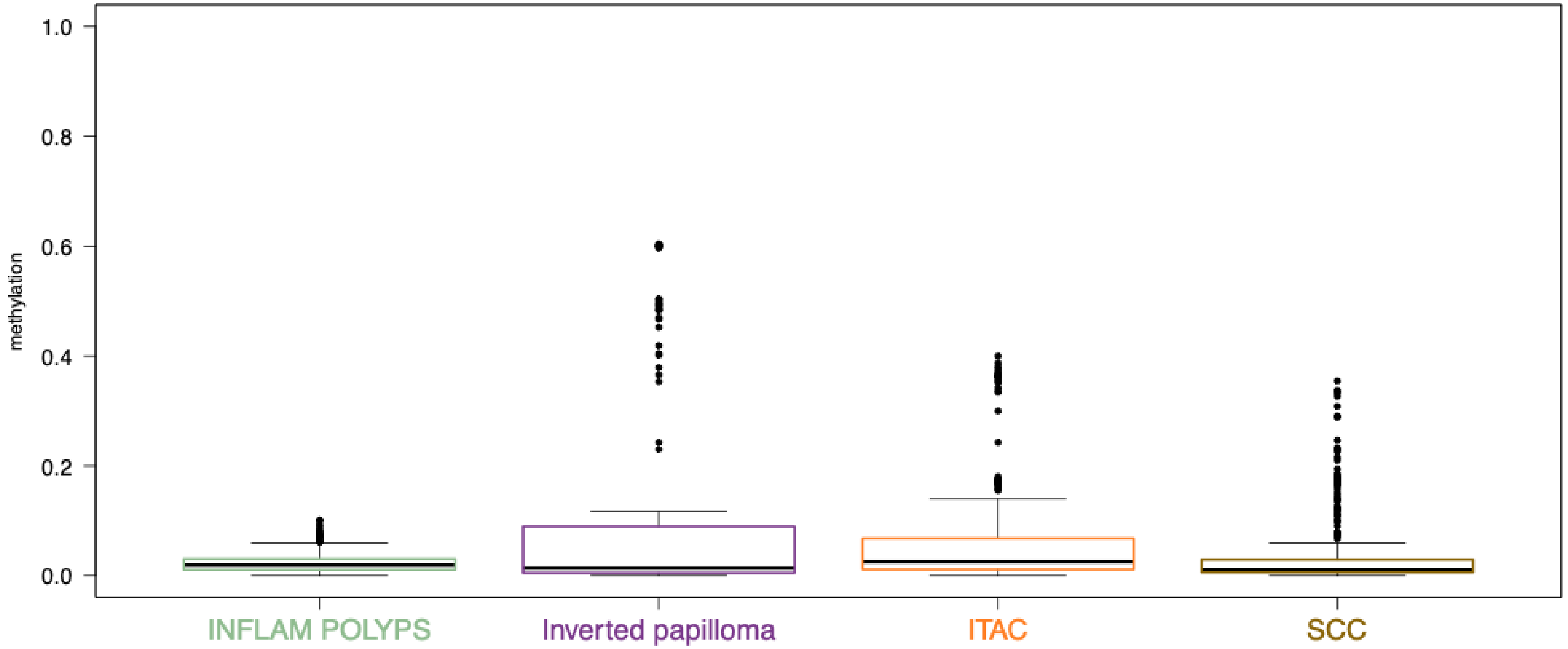
methylation
1.0
0.8
0.6
0.4
0.2
0.0
INFLAM POLYPS
Inverted papilloma
ITAC
SCC

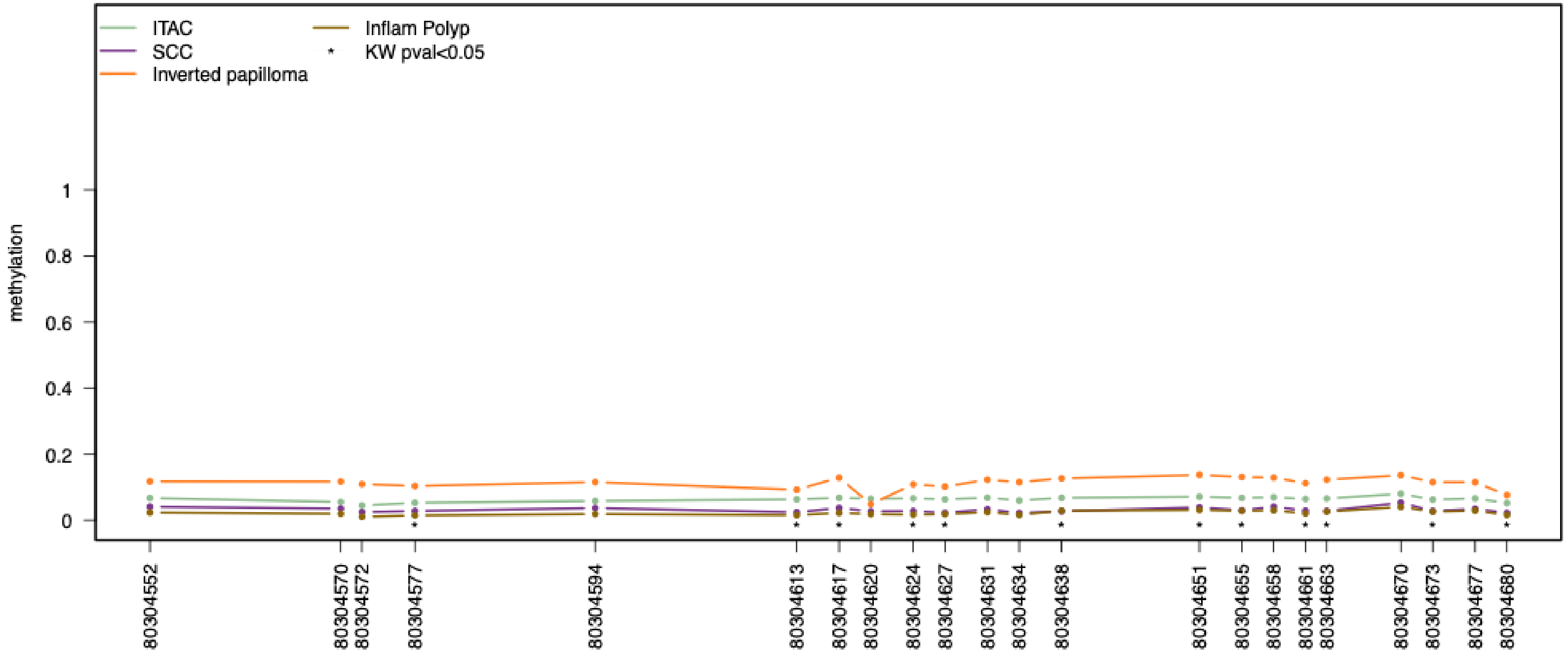
ITAC
SCC
Inverted papilloma
Inflam Polyp
* KW pval<0.05
methylation
1
0.8
0.6
0.4
0.2
0
80304552
80304570
80304572
80304577
80304594
80304613
80304617
80304620
80304624
80304627
80304631
80304634
80304638
80304651
80304655
80304658
80304661
80304663
80304670
80304673
80304677
80304680

LRRTM1

	Position	ITAC Methyla	Inflam Polyp I	Inverted papi	SCC Methyla	ITAC Methyla	Inflam Polyp I	Inverted papi	SCC Methyla	ITAC Methyla	Inflam Polyp I	Inverted papi	SCC Methyla	ITAC Methyla	Inflam Polyp I	Inverted papi	SCC Methyla	ITAC Number	Inflam Polyp I	Inverted papi	SCC Number	Kruskall-Wal
V2	80304552	0.06737143	0.02339722	0.11775833	0.04118621	0.09847756	0.01736904	0.20499864	0.07679354	0.0061	0	0.0005	0.0004	0.4001	0.079	0.5994	0.3352	0	0	0	0	0.06164
V3	80304570	0.05538571	0.01965833	0.11721667	0.03543448	0.08994653	0.01615011	0.20530599	0.07190549	0.0026	0	0.0005	0	0.3425	0.069	0.6015	0.3547	0	0	0	0	0.18958
V4	80304572	0.04493333	0.01044444	0.10948333	0.02482414	0.07409505	0.00949043	0.20685167	0.05811039	0e+00	0e+00	5e-04	0e+00	0.2428	0.0419	0.6	0.2898	0	0	0	0	0.19337
V5	80304577	0.0530619	0.014775	0.1038	0.02818621	0.0855916	0.01291992	0.18878342	0.0670789	0.0017	0	0.0048	0	0.334	0.0547	0.603	0.3327	0	0	0	0	0.03071
V6	80304594	0.05863333	0.01893333	0.11549167	0.03713103	0.09456791	0.01529147	0.20596707	0.07678724	0	0	0	0	0.3628	0.0732	0.6039	0.3372	0	0	0	0	0.20702
V7	80304613	0.06348571	0.01561111	0.0925	0.02441724	0.09635691	0.0151435	0.16939861	0.04741242	0.0006	0	0.0022	0	0.3656	0.0703	0.483	0.1721	0	0	0	0	0.01272
V8	80304617	0.06795714	0.02231111	0.12900833	0.03732414	0.09592634	0.02033508	0.20361596	0.07089918	0.0012	0	0.0011	0	0.3621	0.0912	0.5985	0.2888	0	0	0	0	0.02588
V9	80304620	0.06522381	0.01850278	0.0489	0.0274	0.09499902	0.01682964	0.06989763	0.04514798	6e-04	0e+00	0e+00	1e-03	0.3708	0.0838	0.2426	0.1777	0	0	0	0	0.06545
V10	80304624	0.06648571	0.01720278	0.10871667	0.02805862	0.09842458	0.015862	0.17566545	0.0499484	0	0	0	0	0.3698	0.0678	0.483	0.1856	0	0	0	0	0.01706
V11	80304627	0.06355238	0.01850556	0.102075	0.02276552	0.09649379	0.01898795	0.15762723	0.03896682	0.0015	0	0	0	0.3628	0.0789	0.4844	0.1742	0	0	0	0	0.03065
V12	80304631	0.06838095	0.02463056	0.12286667	0.03347241	0.09555357	0.02161724	0.20194397	0.05683162	0.0015	0.0007	0	0	0.3572	0.0863	0.6	0.2101	0	0	0	0	0.08498
V13	80304634	0.06005238	0.01635	0.1154	0.02200345	0.09315722	0.01509135	0.19939219	0.03460069	6e-04	0e+00	0e+00	0e+00	0.3652	0.0703	0.597	0.1638	0	0	0	0	0.08598
V14	80304638	0.06807619	0.02898333	0.1269	0.02725517	0.09801555	0.02418713	0.20363183	0.04714513	0.0012	0	0	0	0.3804	0.0999	0.6015	0.1763	0	0	0	0	0.01408
V15	80304651	0.0714381	0.03135	0.13753333	0.03933448	0.08760654	0.02412864	0.19831924	0.06815248	0.0049	0.0013	0.0033	0	0.3368	0.0999	0.6015	0.3083	0	0	0	0	0.02514
V16	80304655	0.0678381	0.02859444	0.13114167	0.03007931	0.09639357	0.02535828	0.20064893	0.04651194	0.0012	0	0.0022	0	0.3621	0.1097	0.6	0.1728	0	0	0	0	0.02105
V17	80304658	0.0697381	0.02997778	0.1292	0.04123448	0.09622044	0.02285093	0.20135676	0.06051594	0.0006	0	0.0058	0.0004	0.3768	0.09	0.603	0.2327	0	0	0	0	0.17445
V18	80304661	0.06458571	0.020425	0.11223333	0.02925172	0.09607662	0.01869084	0.18030471	0.05115091	0.0019	0	0	0	0.3544	0.0838	0.5985	0.2152	0	0	0	0	0.04496
V19	80304663	0.06600476	0.02625	0.12295	0.02838276	0.09671287	0.01969706	0.18618544	0.04416053	6e-04	0e+00	0e+00	0e+00	0.364	0.0725	0.5994	0.1812	0	0	0	0	0.047
V20	80304670	0.0803619	0.04062778	0.13653333	0.05328621	0.09864564	0.02664191	0.20031016	0.08157711	0.0012	0	0.0022	0.0018	0.3874	0.1085	0.6	0.3267	0	0	0	0	0.20308
V21	80304673	0.06266667	0.02615833	0.11540833	0.02806207	0.09367568	0.01851352	0.19640552	0.04848868	0	0.0014	0.0044	0	0.3642	0.0726	0.6	0.1791	0	0	0	0	0.01917
V22	80304677	0.06616667	0.02919167	0.11506667	0.03465517	0.09175026	0.02085841	0.18791823	0.05912888	0	0	0.0033	0.0004	0.3516	0.1007	0.6	0.2464	0	0	0	0	0.06686
V23	80304680	0.05205238	0.015975	0.07636667	0.02261379	0.07857691	0.0127871	0.14306296	0.03943858	0.0053	0	0.0016	0.0005	0.2999	0.0424	0.4844	0.1677	0	0	0	0	0.04063

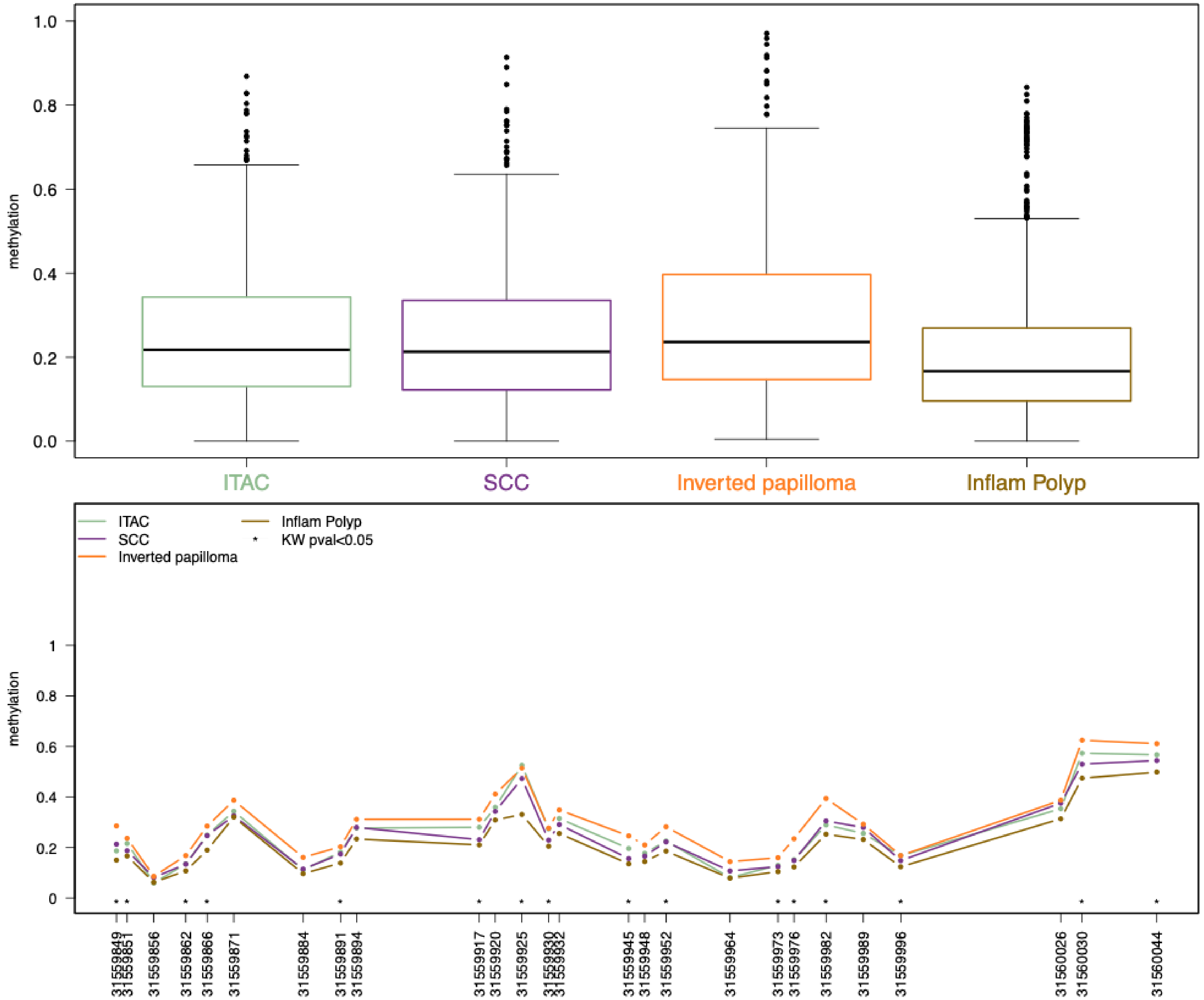

1.0
0.8
0.6
0.4
0.2
0.0
methylation
ITAC
SCC
Inverted papilloma
Inflam Polyp
ITAC
SCC
Inverted papilloma
Inflam Polyp
KW pval<0.05
1
0.8
0.6
0.4
0.2
0
methylation
31559849
31559851
31559856
31559862
31559866
31559871
31559884
31559891
31559894
31559917
31559920
31559925
31559930
31559932
31559945
31559948
31559952
31559964
31559973
31559976
31559982
31559989
31559996
31560026
31560030
31560044

MIR193

MIR193

	Position	ITAC Methyla	Inflam Polyp I	Inverted papi	SCC Methyla	ITAC Methyla	Inflam Polyp I	Inverted papi	SCC Methyla	ITAC Methyla	Inflam Polyp I	Inverted papi	SCC Methyla	ITAC Methyla	Inflam Polyp I	Inverted papi	SCC Methyla	ITAC Number	Inflam Polyp I	Inverted papi	SCC Number	Kruskall-Wal
V2	31559849	0.1865619	0.150125	0.2854333	0.2132103	0.05859428	0.11535602	0.24171291	0.12217103	0.0658	0.013	0.1073	0	0.2813	0.715	0.9133	0.5608	0	0	0	0	0.001486
V3	31559851	0.2160524	0.1664056	0.236475	0.1872414	0.1300149	0.1237966	0.1230457	0.1232761	0.0823	0.0389	0.1097	0	0.7239	0.7278	0.5685	0.4986	0	0	0	0	0.0224087
V4	31559856	0.0599381	0.06244444	0.08608333	0.08164483	0.04144453	0.1082812	0.07751888	0.07439552	0	0.0094	0.0047	0	0.1592	0.679	0.2847	0.2666	0	0	0	0	0.1175948
V5	31559862	0.1354333	0.1076611	0.1680667	0.1342966	0.11257041	0.10990156	0.09626948	0.09850605	0	0.0295	0.0877	0	0.5153	0.7207	0.4545	0.371	0	0	0	0	0.0084997
V6	31559866	0.2491095	0.1896417	0.2846667	0.2470379	0.1178089	0.1156046	0.1577219	0.1050403	0.0813	0.0802	0.1451	0.0057	0.5247	0.7245	0.6434	0.5163	0	0	0	0	0.0019173
V7	31559871	0.3434	0.3204833	0.3865667	0.3253655	0.1108208	0.1067186	0.1286402	0.1267937	0.1116	0.1962	0.237	0.0109	0.6012	0.7621	0.7339	0.587	0	0	0	0	0.1407768
V8	31559884	0.1110381	0.09644722	0.16095	0.11484138	0.08167778	0.10565756	0.14661949	0.07264071	0.004	0.0132	0.0442	0.0003	0.3673	0.6767	0.5013	0.2806	0	0	0	0	0.1200444
V9	31559891	0.1819905	0.1386583	0.203025	0.1739483	0.1067543	0.1079703	0.1550595	0.1020427	0.095	0.0493	0.0919	0	0.5951	0.6966	0.6301	0.4244	0	0	0	0	0.0070509
V10	31559894	0.2768381	0.2337139	0.3116917	0.2793724	0.1043765	0.1086252	0.1512858	0.1105262	0.1547	0.0943	0.1665	0.129	0.6196	0.7429	0.6817	0.5483	0	0	0	0	0.0686585
V11	31559917	0.2799857	0.2101972	0.3119167	0.2309828	0.1274889	0.1149188	0.2236148	0.1058472	0.1144	0.0681	0.0568	0.0323	0.5951	0.7497	0.8815	0.4599	0	0	0	0	0.0249725
V12	31559920	0.3593238	0.3089222	0.4113083	0.3428655	0.1057033	0.1181015	0.1814026	0.1267766	0.145	0.131	0.2082	0.019	0.5912	0.7795	0.7788	0.6275	0	0	0	0	0.0601176
V13	31559925	0.5253476	0.3309889	0.5137333	0.4723828	0.1286667	0.1686123	0.2083467	0.2119605	0.2856	0.1259	0.2568	0.0664	0.737	0.7795	0.9451	0.914	0	0	0	0	0.0002864
V14	31559930	0.2737857	0.2050583	0.2767667	0.2292483	0.1252438	0.1128741	0.1509197	0.1070016	0.1123	0.0961	0.1516	0.0215	0.6012	0.7573	0.7082	0.5089	0	0	0	0	0.0425539
V15	31559932	0.314881	0.2555944	0.3487417	0.2909345	0.12356	0.1125533	0.1998389	0.1129598	0.0995	0.1088	0.1688	0.0531	0.5988	0.763	0.7977	0.5448	0	0	0	0	0.0863382
V16	31559945	0.1961381	0.1355167	0.246125	0.156231	0.10102529	0.11170426	0.25415114	0.08660012	0.0756	0.029	0.0599	0.0108	0.5337	0.7103	0.9191	0.4398	0	0	0	0	0.0063197
V17	31559948	0.176919	0.1445806	0.2100667	0.1656552	0.1135452	0.1180363	0.1225951	0.1028717	0.0446	0.0217	0.0509	0.0152	0.5706	0.7474	0.5151	0.4532	0	0	0	0	0.1092829
V18	31559952	0.2256381	0.1856639	0.2819333	0.2222552	0.07763588	0.11766262	0.1611239	0.1181178	0.0798	0.0304	0.15	0	0.4064	0.7532	0.677	0.5355	0	0	0	0	0.0114756
V19	31559964	0.08101429	0.07873611	0.14423333	0.10731724	0.04908353	0.11104166	0.19251855	0.09049963	0	0	0.0315	0.0007	0.1643	0.7062	0.5983	0.4743	0	0	0	0	0.1421313
V20	31559973	0.1294857	0.1047222	0.15975	0.1243483	0.1042259	0.1156423	0.1061682	0.0948902	0	0.0179	0.0707	0	0.4969	0.737	0.4721	0.4539	0	0	0	0	0.0181695
V21	31559976	0.1515905	0.1229222	0.234575	0.1489069	0.09443923	0.11275017	0.21340783	0.0958639	0	0.0355	0.0784	0.0065	0.3743	0.7345	0.7457	0.4622	0	0	0	0	0.0415501
V22	31559982	0.2893619	0.2527083	0.3939167	0.3050034	0.1227585	0.1391985	0.1850975	0.1410344	0.0184	0.0879	0.1812	0	0.5363	0.7704	0.8179	0.6275	0	0	0	0	0.0095708
V23	31559989	0.2557667	0.2316167	0.2922333	0.2791966	0.111337	0.104968	0.1258032	0.1073486	0.0245	0.0962	0.1567	0.0215	0.4627	0.7334	0.6393	0.5363	0	0	0	0	0.0606361
V24	31559996	0.1679429	0.1237333	0.1681667	0.1482655	0.1033422	0.11275928	0.13240136	0.08507427	0.0459	0.0215	0.0615	0	0.5215	0.7423	0.5619	0.3678	0	0	0	0	0.0214102
V25	31560026	0.3534857	0.3136306	0.3869333	0.3754069	0.1490309	0.1053028	0.1249413	0.1262214	0.1254	0.1665	0.207	0.2046	0.8037	0.8097	0.6687	0.6733	0	0	0	0	0.0562823
V26	31560030	0.5730286	0.4740333	0.6240417	0.5299724	0.1410321	0.1298317	0.1669082	0.1497306	0.2881	0.2627	0.3949	0.186	0.8282	0.8425	0.9594	0.8904	0	0	0	0	0.0095613
V27	31560044	0.566819	0.4982722	0.6107417	0.5439034	0.1484021	0.1130312	0.167257	0.1365967	0.2259	0.2989	0.4031	0.2015	0.8685	0.8258	0.9709	0.8494	0	0	0	0	0.0200486

MIR296

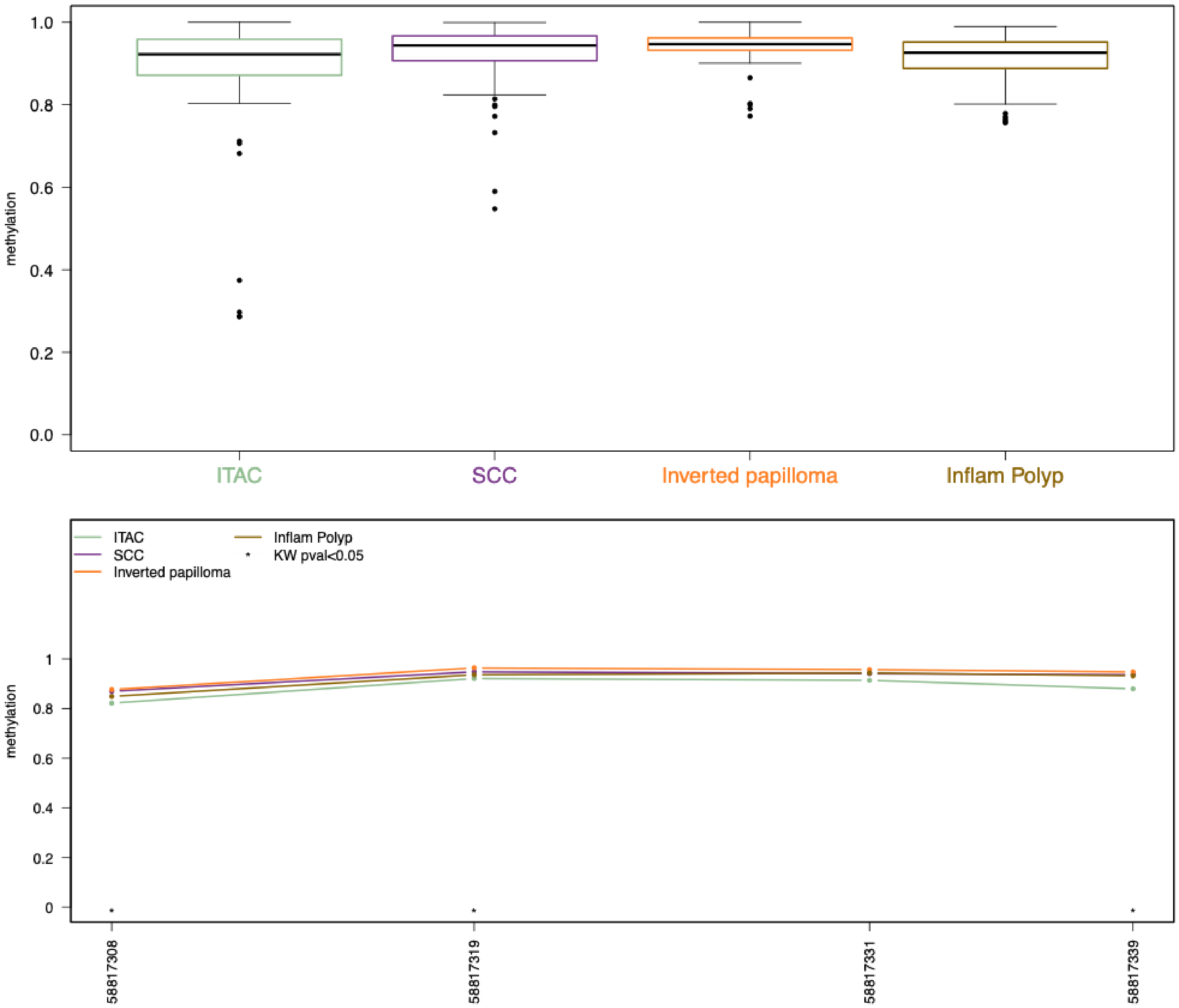

methylation
1.0
0.8
0.6
0.4
0.2
0.0
ITAC
SCC
Inverted papilloma
Inflam Polyp
ITAC
SCC
Inverted papilloma
Inflam Polyp
KW pval<0.05
methylation
1
0.8
0.6
0.4
0.2
0
58817308
58817319
58817331
58817339

MIR296

	Position	ITAC Methyla	Inflam Polyp I	Inverted papi	SCC Methyla	ITAC Methyla	Inflam Polyp I	Inverted papi	SCC Methyla	ITAC Methyla	Inflam Polyp I	Inverted papi	SCC Methyla	ITAC Methyla	Inflam Polyp I	Inverted papi	SCC Methyla	ITAC Number	Inflam Polyp I	Inverted papi	SCC Number	Kruskall-Wal
V2	58817308	0.8216238	0.848875	0.8761667	0.8695138	0.14233502	0.04524575	0.06976321	0.09430162	0.297	0.7563	0.7727	0.5477	1	0.9351	1	0.9688	0	0	0	0	0.038529
V3	58817319	0.9211095	0.9357917	0.9638	0.9478517	0.13170828	0.03134179	0.01995285	0.0434193	0.374	0.8608	0.9344	0.7721	1	0.9899	1	0.9993	0	0	0	0	0.037719
V4	58817331	0.9136	0.9430889	0.9568833	0.9406448	0.14741435	0.02609504	0.01824614	0.05409783	0.287	0.8514	0.9333	0.7323	1	0.9806	1	0.9848	0	0	0	0	0.476724
V5	58817339	0.8793429	0.93185	0.9471667	0.9361724	0.14314168	0.02115944	0.02137545	0.04000204	0.286	0.8728	0.9092	0.7994	0.9727	0.9705	1	0.9972	0	0	0	0	0.005846

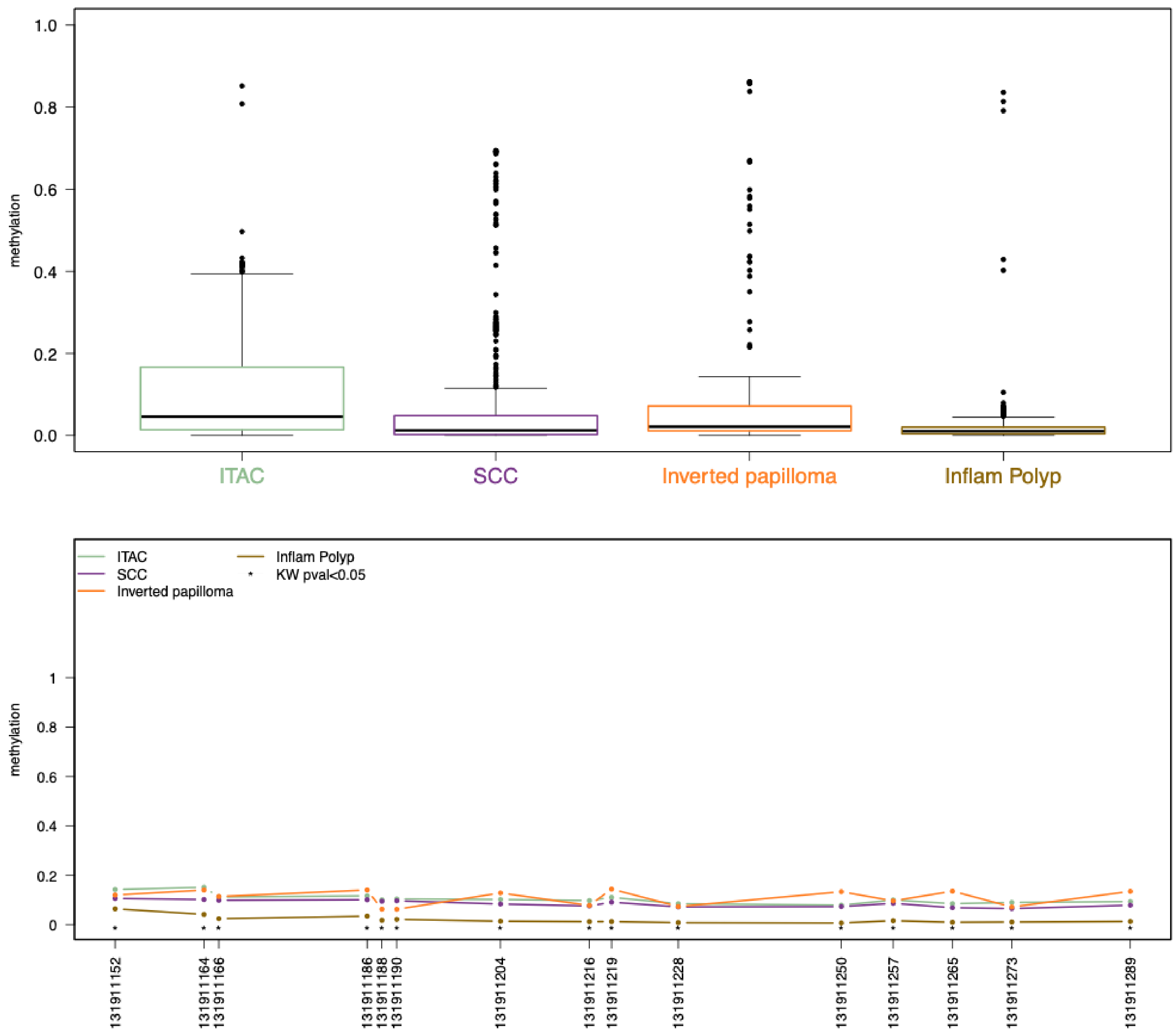

1.0
0.8
0.6
0.4
0.2
0.0
methylation
ITAC
SCC
Inverted papilloma
Inflam Polyp
ITAC
SCC
Inverted papilloma
Inflam Polyp
KW pval<0.05
1
0.8
0.6
0.4
0.2
0
methylation
131911152
131911164
131911166
131911186
131911188
131911190
131911204
131911216
131911219
131911228
131911250
131911257
131911265
131911273
131911289

NTM

NTM

	Position	ITAC Methyla	Inflam Polyp	Inverted papi	SCC Methyla	ITAC Methyla	Inflam Polyp	Inverted papi	SCC Methyla	ITAC Methyla	Inflam Polyp	Inverted papi	SCC Methyla	ITAC Methyla	Inflam Polyp	Inverted papi	SCC Methyla	ITAC Number	Inflam Polyp	Inverted papi	SCC Number	Kruskall-Wal
V2	131911152	0.14258571	0.06414444	0.12010833	0.10613448	0.2034573	0.1880312	0.2099442	0.1918167	0.0062	0.0006	0.0035	0	0.852	0.836	0.666	0.6946	0	0	0	0	0.0193977
V3	131911164	0.15174762	0.04148056	0.140425	0.10189655	0.2025042	0.1295878	0.2565975	0.1854933	0	0	0.0014	0	0.808	0.7913	0.8619	0.6924	0	0	0	0	0.0082632
V4	131911166	0.11218571	0.0243	0.11389167	0.09888621	0.13076235	0.06574936	0.23951423	0.18230601	0.0037	0	0.0049	0	0.4157	0.4023	0.8384	0.6126	0	0	0	0	0.003604
V5	131911186	0.11652857	0.03436667	0.14063333	0.10102069	0.13130043	0.06923225	0.23463577	0.18374499	0	0.0004	0.0014	0	0.4157	0.4288	0.6701	0.6917	0	0	0	0	0.0125543
V6	131911188	0.10048571	0.01788611	0.06279167	0.09483448	0.12142952	0.01524827	0.11341156	0.17901495	0	0.0006	0.0021	0	0.4157	0.0602	0.4022	0.6885	0	0	0	0	0.0343392
V7	131911190	0.10412381	0.02146389	0.06235833	0.09672759	0.12389543	0.01854947	0.09818904	0.1772243	0	0	0.0091	0	0.4236	0.071	0.3501	0.6863	0	0	0	0	0.0101589
V8	131911204	0.10207143	0.01398056	0.12855	0.08382069	0.12436453	0.01536766	0.23082741	0.17158621	0.0031	0.0004	0	0	0.4157	0.0635	0.668	0.6917	0	0	0	0	0.0014299
V9	131911216	0.09757619	0.01226389	0.07799167	0.07592069	0.131413	0.0173552	0.1266329	0.1661124	0e+00	0e+00	7e-04	0e+00	0.4157	0.0792	0.4228	0.6618	0	0	0	0	0.0043776
V10	131911219	0.11038571	0.01242222	0.14429167	0.09122414	0.14243919	0.01449641	0.27707504	0.18015854	0.0031	0	0.0031	0	0.4969	0.0622	0.8621	0.6936	0	0	0	0	0.0007964
V11	131911228	0.08495714	0.00806111	0.07350833	0.07203103	0.12652061	0.01312022	0.12620658	0.14443042	0	0	0.0014	0	0.4323	0.0646	0.4233	0.5678	0	0	0	0	0.001527
V12	131911250	0.07921429	0.00678333	0.133575	0.0732	0.10707107	0.01083933	0.28121785	0.15562473	0e+00	0e+00	7e-04	0e+00	0.4116	0.0531	0.8589	0.6296	0	0	0	0	0.0002906
V13	131911257	0.10007143	0.016125	0.09635	0.08670345	0.12182224	0.01384253	0.16313026	0.1715717	0.0025	0	0	0	0.4157	0.0656	0.5508	0.6915	0	0	0	0	0.0039482
V14	131911265	0.08518095	0.00951667	0.13575	0.06873448	0.12835046	0.01287197	0.27032344	0.14520981	0	0	0	0	0.4199	0.0619	0.8599	0.5376	0	0	0	0	0.0042742
V15	131911273	0.09011905	0.01058611	0.07183333	0.06517586	0.1208321	0.0121843	0.1158041	0.1221335	0	0	0.0072	0	0.4157	0.0619	0.3878	0.457	0	0	0	0	0.0001511
V16	131911289	0.09370476	0.01298889	0.13498333	0.0789	0.12093314	0.01262007	0.2661119	0.15180727	0.0032	0	0	0	0.4182	0.0669	0.8573	0.6601	0	0	0	0	0.0035282

PARP15

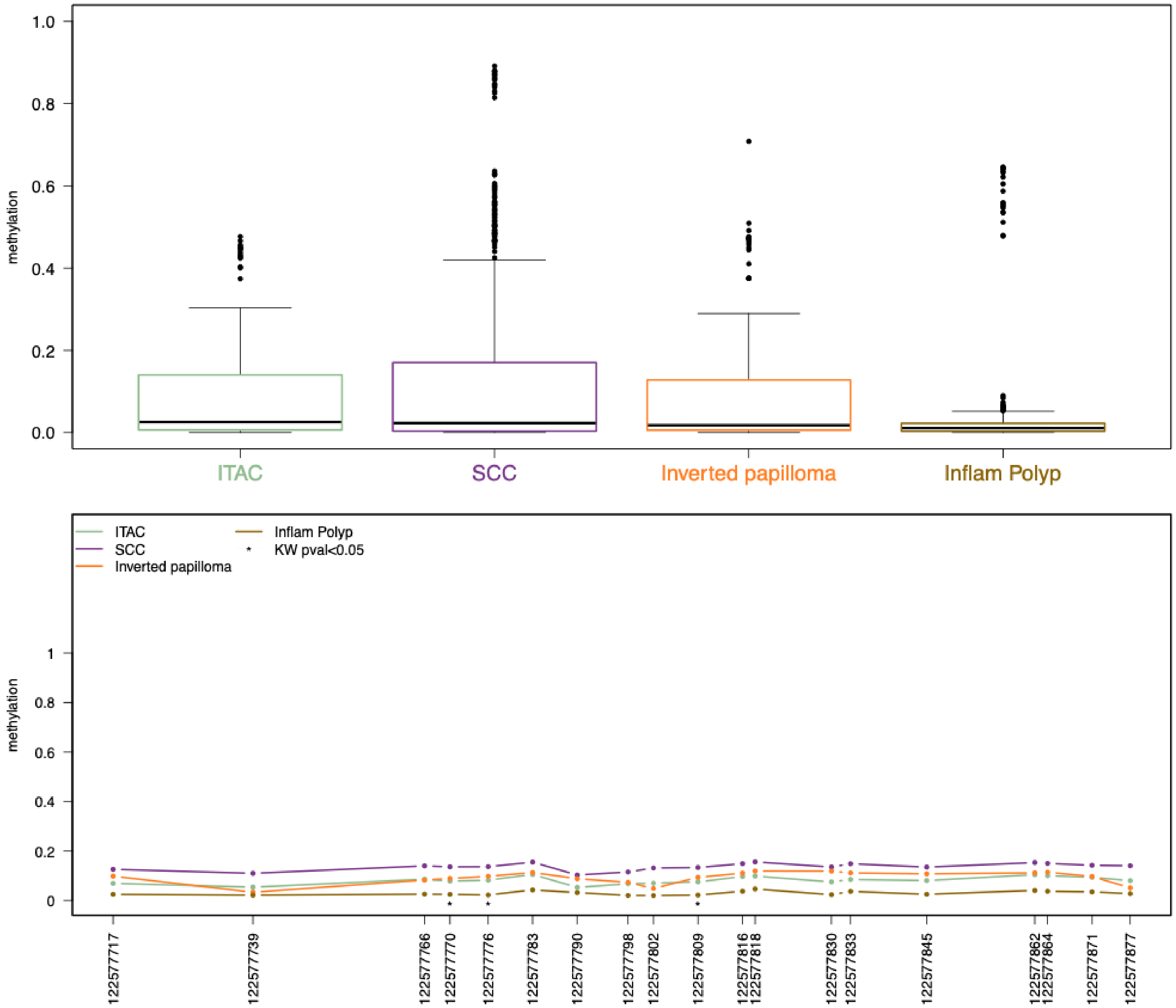

methylation
1.0
0.8
0.6
0.4
0.2
0.0
ITAC
SCC
Inverted papilloma
Inflam Polyp
ITAC
SCC
Inverted papilloma
Inflam Polyp
KW pval<0.05
methylation
1
0.8
0.6
0.4
0.2
0
122577717
122577739
122577766
122577770
122577776
122577783
122577790
122577798
122577802
122577809
122577816
122577818
122577830
122577833
122577845
122577862
122577864
122577871
122577877

PARP15

	Position	ITAC Methyla	Inflam Polyp I	Inverted papi	SCC Methyla	ITAC Methyla	Inflam Polyp I	Inverted papi	SCC Methyla	ITAC Methyla	Inflam Polyp I	Inverted papi	SCC Methyla	ITAC Methyla	Inflam Polyp I	Inverted papi	SCC Methyla	ITAC Number	Inflam Polyp I	Inverted papi	SCC Number	Kruskall-Wal
V2	122577717	0.0691	0.02479167	0.09828333	0.12599655	0.09090317	0.09183137	0.20593068	0.2092133	0	0	0.0015	0	0.2903	0.5588	0.7083	0.8145	0	0	0	0	0.05354
V3	122577739	0.05380952	0.02147222	0.03335	0.10973448	0.08435583	0.0922546	0.04815442	0.20276182	0	0	0	0	0.2959	0.5588	0.1367	0.8258	0	0	0	0	0.30102
V4	122577766	0.08601429	0.02554444	0.08279167	0.13994483	0.11285528	0.09066203	0.13385503	0.23154159	0	0	0.0023	0	0.4297	0.5508	0.4499	0.8773	0	0	0	0	0.08403
V5	122577770	0.07851429	0.02465278	0.08915	0.13601724	0.10850367	0.09985457	0.14223995	0.23019275	0	0	0	0	0.4034	0.6047	0.4725	0.8776	0	0	0	0	0.04643
V6	122577776	0.08215714	0.02217778	0.09770833	0.13672414	0.11518666	0.07855253	0.14721263	0.22865353	0	0	0	0	0.4511	0.4779	0.4682	0.84	0	0	0	0	0.03767
V7	122577783	0.10539048	0.04305556	0.112925	0.15513793	0.1163187	0.1026932	0.1474927	0.2291413	0	0	0.0069	0.0024	0.4518	0.6326	0.4576	0.8719	0	0	0	0	0.07997
V8	122577790	0.05282857	0.031925	0.0881	0.10274138	0.07358323	0.10434872	0.14518708	0.18814443	0	0	0	0	0.2761	0.6366	0.473	0.8478	0	0	0	0	0.39012
V9	122577798	0.06748095	0.02030833	0.07329167	0.11511379	0.10222692	0.07938693	0.12872795	0.20418764	0	0	0	0	0.4002	0.4798	0.4445	0.8309	0	0	0	0	0.21612
V10	122577802	0.07013333	0.01998056	0.04859167	0.13135862	0.09954986	0.08848424	0.07617921	0.22480488	0	0	0	0	0.3738	0.5348	0.25	0.8435	0	0	0	0	0.06941
V11	122577809	0.07505714	0.02184167	0.09338333	0.13327931	0.11431688	0.08851815	0.14520982	0.2277677	0e+00	0e+00	8e-04	0e+00	0.448	0.5359	0.4712	0.8607	0	0	0	0	0.02933
V12	122577816	0.09697619	0.0375	0.11044167	0.14902414	0.1164108	0.1046684	0.1550859	0.2281174	0	0	0.0023	0	0.4451	0.6429	0.4748	0.8578	0	0	0	0	0.12971
V13	122577818	0.09857143	0.04664722	0.11903333	0.15604138	0.116727	0.1045211	0.1524231	0.2272945	0	0	0.0053	0.0017	0.4566	0.6433	0.4709	0.8697	0	0	0	0	0.18117
V14	122577830	0.07509048	0.02338333	0.11854167	0.13532414	0.11087993	0.08991814	0.17123954	0.23136804	0	0	0	0	0.4336	0.5462	0.4735	0.8467	0	0	0	0	0.20865
V15	122577833	0.08520952	0.03680278	0.11135833	0.14868276	0.1172946	0.101865	0.1578496	0.2276086	0e+00	0e+00	0e+00	9e-04	0.4471	0.6218	0.4758	0.8654	0	0	0	0	0.30607
V16	122577845	0.08069524	0.02499444	0.107625	0.13503103	0.12094363	0.08395804	0.16085402	0.22176288	0	0	0	0	0.4663	0.5115	0.4634	0.8613	0	0	0	0	0.10435
V17	122577862	0.10444762	0.04093611	0.1117	0.15335517	0.1139705	0.1044023	0.1679206	0.2354676	0e+00	0e+00	8e-04	0e+00	0.4388	0.6428	0.5094	0.88	0	0	0	0	0.0543
V18	122577864	0.10014286	0.03734167	0.11406667	0.15003793	0.12034355	0.09565554	0.16967853	0.23856921	0	0	0.0021	0	0.4766	0.5875	0.4915	0.8916	0	0	0	0	0.21855
V19	122577871	0.09371429	0.03485	0.09708333	0.14231379	0.1120898	0.1058567	0.1464898	0.2280011	0	0	0.0016	0	0.4237	0.646	0.4104	0.8798	0	0	0	0	0.08399
V20	122577877	0.08015714	0.02754444	0.05090833	0.1404069	0.1152895	0.09055564	0.07698036	0.23470687	0	0	0	0	0.4267	0.553	0.25	0.8718	0	0	0	0	0.31954

TERT

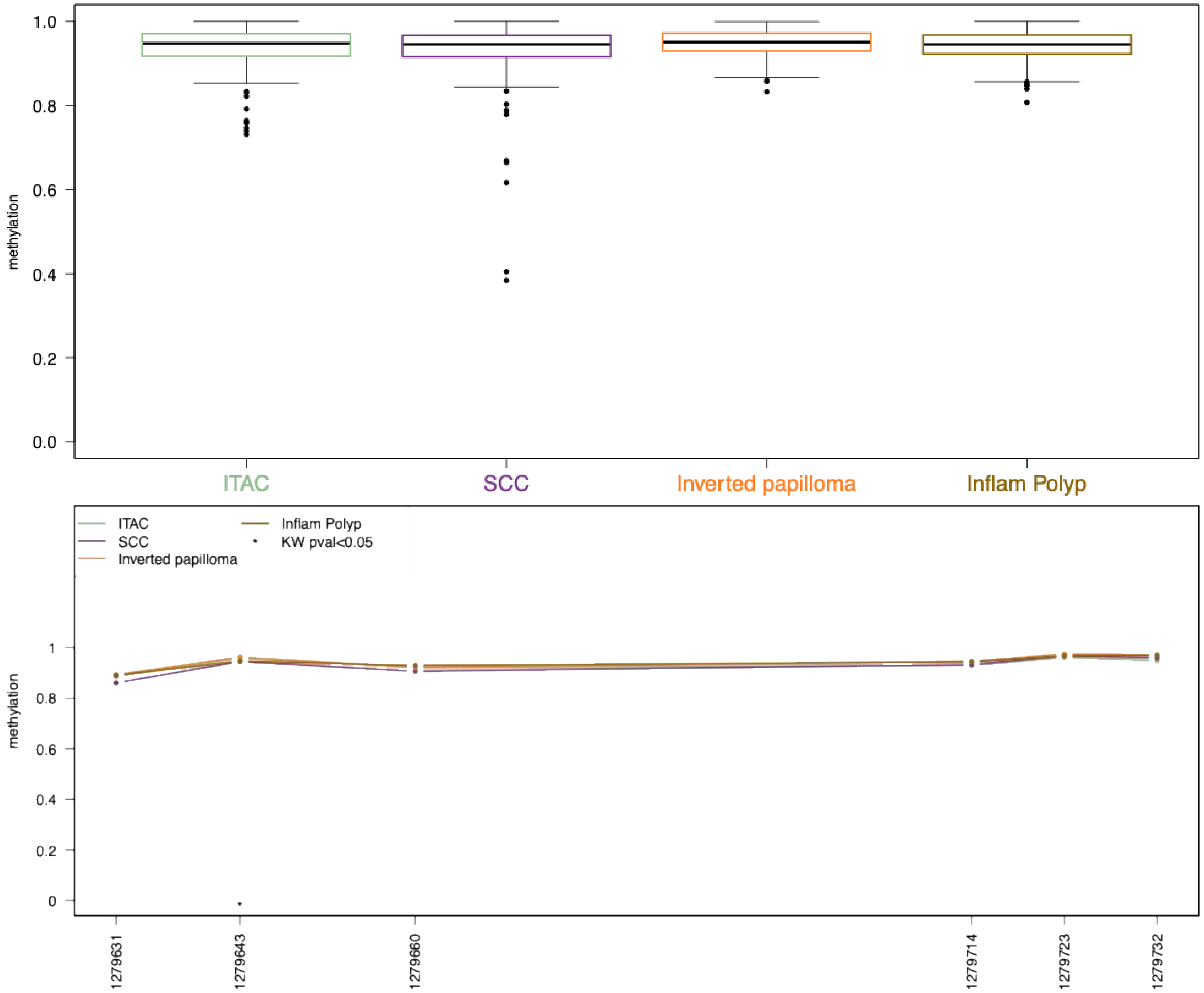

methylation
1.0
0.8
0.6
0.4
0.2
0.0
ITAC
SCC
Inverted papilloma
Inflam Polyp
ITAC
SCC
Inverted papilloma
Inflam Polyp
KW pval<0.05
methylation
1
0.8
0.6
0.4
0.2
0
1279631
1279643
1279660
1279714
1279723
1279732

TERT

	Position	ITAC Methyla	Inflam Polyp I	Inverted papi	SCC Methyla	ITAC Methyla	Inflam Polyp I	Inverted papi	SCC Methyla	ITAC Methyla	Inflam Polyp I	Inverted papi	SCC Methyla	ITAC Methyla	Inflam Polyp I	Inverted papi	SCC Methyla	ITAC Number	Inflam Polyp I	Inverted papi	SCC Number	Kruskall-Wal
V2	1279631	0.8860714	0.8898083	0.892675	0.8602828	0.06008688	0.02419617	0.02805463	0.13922827	0.7314	0.8402	0.8576	0.3841	0.9717	0.9412	0.9335	0.964	0	0	0	0	0.81469
V3	1279643	0.9580714	0.9457667	0.961825	0.9439172	0.04376879	0.031414	0.01895862	0.03685749	0.7921	0.8492	0.9362	0.8347	1	0.9979	0.999	0.993	0	0	0	0	0.04429
V4	1279660	0.9202143	0.9293722	0.921325	0.9059517	0.05247247	0.02953405	0.03711976	0.0823662	0.7471	0.8078	0.8332	0.6163	1	0.9979	0.9702	0.9802	0	0	0	0	0.94931
V5	1279714	0.929	0.943175	0.9452	0.9317828	0.04914054	0.0173199	0.02195798	0.04692921	0.7395	0.9083	0.9076	0.7791	0.9804	0.9798	0.9942	0.982	0	0	0	0	0.81009
V6	1279723	0.9599095	0.9671861	0.9744417	0.9669	0.04770851	0.015378	0.01237347	0.01903007	0.7647	0.9362	0.9583	0.9026	1	1	0.9993	1	0	0	0	0	0.53867
V7	1279732	0.9474143	0.9681	0.9711333	0.9584828	0.05539987	0.01307254	0.01759935	0.02225307	0.7595	0.9331	0.9432	0.8993	0.998	1	0.998	0.9994	0	0	0	0	0.13444

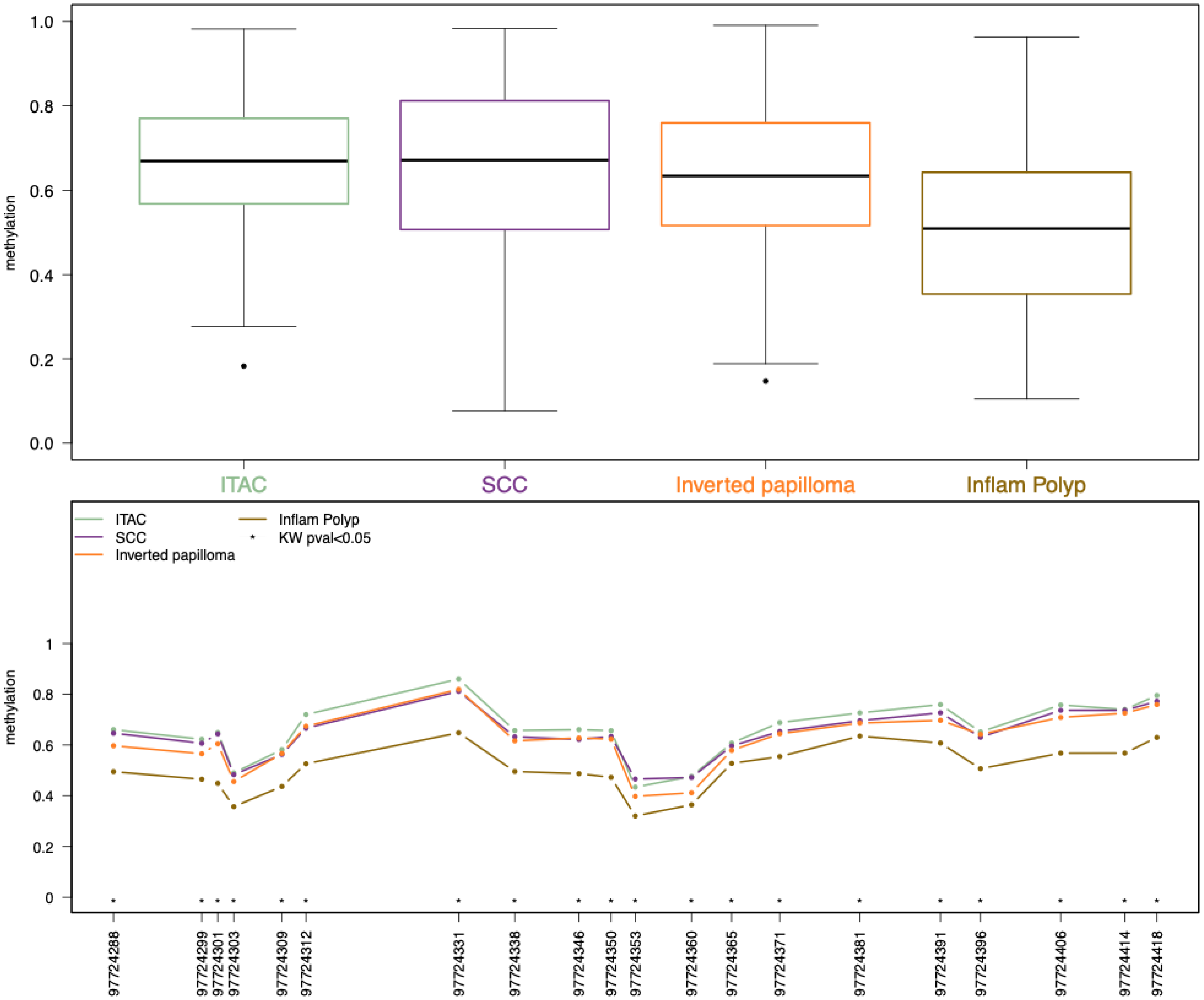

1.0
0.8
0.6
0.4
0.2
0.0
methylation
ITAC
SCC
Inverted papilloma
Inflam Polyp
ITAC
SCC
Inverted papilloma
Inflam Polyp
KW pval<0.05
1
0.8
0.6
0.4
0.2
0
methylation
97724288
97724299
97724301
97724303
97724309
97724312
97724331
97724338
97724346
97724350
97724353
97724360
97724365
97724371
97724381
97724391
97724396
97724406
97724414
97724418

ZAP70

ZAP70

	Position	ITAC Methyla	Inflam Polyp I	Inverted papi	SCC Methyla	ITAC Methyla	Inflam Polyp I	Inverted papi	SCC Methyla	ITAC Methyla	Inflam Polyp I	Inverted papi	SCC Methyla	ITAC Methyla	Inflam Polyp I	Inverted papi	SCC Methyla	ITAC Number	Inflam Polyp I	Inverted papi	SCC Number	Kruskall-Wal
V2	97724288	0.6602714	0.4946028	0.5966583	0.6464034	0.1090645	0.1596963	0.1624244	0.2017443	0.3931	0.2199	0.293	0.2161	0.7987	0.8187	0.8514	0.9791	0	0	0	0	3.571e-04
V3	97724299	0.6223429	0.4647028	0.5652167	0.6064448	0.1330233	0.1741908	0.1502596	0.208985	0.3352	0.1664	0.2585	0.1684	0.8184	0.8272	0.8033	0.9587	0	0	0	0	4.719e-03
V4	97724301	0.6489619	0.4487667	0.6045917	0.6430172	0.1339448	0.2060834	0.1936179	0.2348265	0.311	0.1049	0.1948	0.1081	0.8453	0.8477	0.8858	0.9664	0	0	0	0	3.635e-04
V5	97724303	0.4899095	0.3568333	0.4556583	0.4828414	0.1130703	0.131745	0.117142	0.1847481	0.1828	0.1388	0.1991	0.1355	0.6756	0.7559	0.643	0.9267	0	0	0	0	8.205e-04
V6	97724309	0.5813905	0.4362583	0.5663667	0.5625828	0.1056647	0.1375524	0.1660494	0.185074	0.3657	0.2155	0.2499	0.1868	0.7577	0.782	0.863	0.9529	0	0	0	0	5.800e-04
V7	97724312	0.7197952	0.5260361	0.673675	0.6661138	0.1364032	0.1892468	0.1631415	0.219318	0.3081	0.1748	0.2524	0.1204	0.8958	0.8468	0.9135	0.9835	0	0	0	0	5.000e-04
V8	97724331	0.8600619	0.6480778	0.8192833	0.8111	0.09543841	0.19498531	0.16550772	0.19519951	0.6388	0.3199	0.3567	0.336	0.9822	0.9635	0.9909	0.9801	0	0	0	0	5.663e-05
V9	97724338	0.6567095	0.4953056	0.6160083	0.6322172	0.1093264	0.1638436	0.1592773	0.1939444	0.3695	0.2271	0.2501	0.2124	0.8152	0.8099	0.844	0.9738	0	0	0	0	1.157e-03
V10	97724346	0.6603429	0.486825	0.6275	0.6216103	0.09875332	0.16201963	0.17200095	0.21655966	0.4793	0.1836	0.2243	0.1444	0.8236	0.8079	0.8718	0.9725	0	0	0	0	6.994e-04
V11	97724350	0.6561333	0.4723583	0.6228583	0.633169	0.1250046	0.1780442	0.1894688	0.21982	0.2812	0.1678	0.2176	0.1896	0.8511	0.8119	0.888	0.9433	0	0	0	0	6.587e-04
V12	97724353	0.4337857	0.3200944	0.3972417	0.4655793	0.1106459	0.121399	0.1367987	0.2053599	0.2791	0.1514	0.1885	0.0762	0.6173	0.7539	0.5978	0.9479	0	0	0	0	1.483e-03
V13	97724360	0.4758619	0.3642139	0.411625	0.4717517	0.1130458	0.153413	0.168112	0.2120231	0.2767	0.1071	0.1475	0.0853	0.6883	0.7738	0.6399	0.9606	0	0	0	0	2.711e-02
V14	97724365	0.6072714	0.5274222	0.5785083	0.5958103	0.08377699	0.12607996	0.13274504	0.17248781	0.4772	0.3103	0.3568	0.2455	0.7872	0.8461	0.7578	0.9375	0	0	0	0	4.654e-02
V15	97724371	0.6879762	0.5541417	0.643875	0.6531069	0.09543198	0.15838565	0.15887489	0.20385363	0.4904	0.2627	0.2826	0.1776	0.8364	0.8706	0.8641	0.9479	0	0	0	0	7.988e-03
V16	97724381	0.7265143	0.6348278	0.68615	0.6955483	0.07993275	0.13256406	0.12695317	0.17008857	0.5512	0.3687	0.4692	0.3249	0.8515	0.8909	0.8427	0.9829	0	0	0	0	4.460e-02
V17	97724391	0.7591667	0.6076278	0.697025	0.7267793	0.1045342	0.1546217	0.1670638	0.1980768	0.549	0.2965	0.3579	0.3249	0.9179	0.8909	0.9294	0.9695	0	0	0	0	1.923e-03
V18	97724396	0.6512857	0.506475	0.6417833	0.6303517	0.09462442	0.16280912	0.15459535	0.19867149	0.457	0.1678	0.3148	0.1774	0.822	0.8298	0.8759	0.9653	0	0	0	0	1.441e-03
V19	97724406	0.7577381	0.5676361	0.7084167	0.7365828	0.1175932	0.2065057	0.1659769	0.206791	0.4453	0.2117	0.2599	0.2656	0.9314	0.901	0.9432	0.9714	0	0	0	0	1.006e-03
V20	97724414	0.7396952	0.5678306	0.72535	0.7364724	0.1243648	0.1983723	0.1736718	0.1944866	0.4049	0.2009	0.2824	0.2695	0.9002	0.8978	0.933	0.9733	0	0	0	0	7.123e-04
V21	97724418	0.7951095	0.6296972	0.7593	0.7721862	0.1031795	0.1853889	0.1544866	0.2017688	0.5159	0.2532	0.3419	0.2072	0.9466	0.918	0.9358	0.9815	0	0	0	0	1.041e-03
