# Supplementary figures and images for: DNA methylation analysis with nasal brushing for early diagnosis of sinonasal malignant tumours
Source: Discov Oncol. 2026 Jan 29;17:357. doi: 10.1007/s12672-026-04508-0 (PMC12923719; doi:10.1007/s12672-026-04508-0)

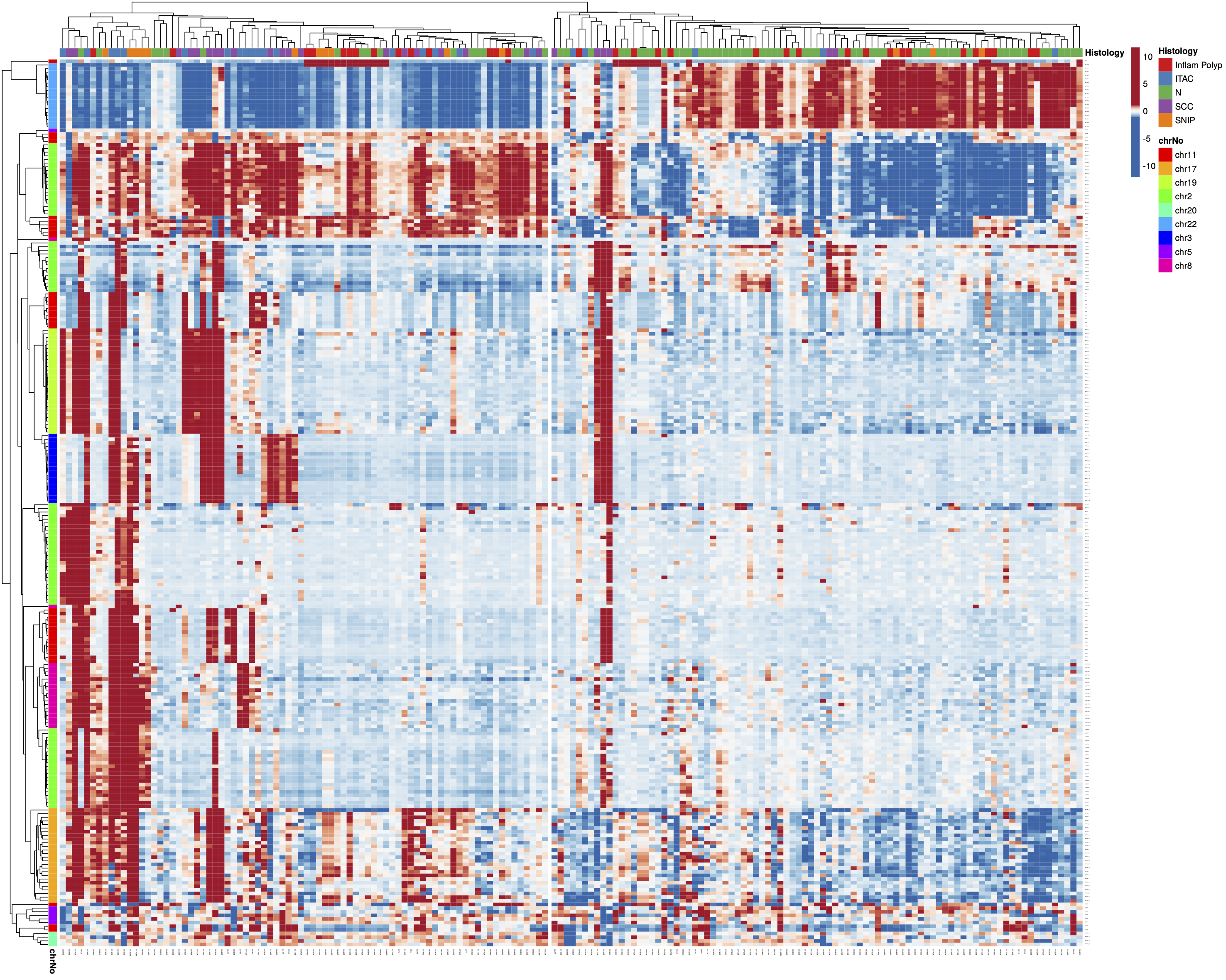

Supplement: Supplementary file 2 — Supplementary Material 2. HeatMap considering all cases in which rows are centered; unit variance scaling is applied to rows. Both rows and columns are clustered using correlation distance and average linkage with 245 rows, 167 columns. Two clusters are marked: right cluster showed 54 normal samples, 19 inflammatory polyps, 6 tumours, 5 ITAC and 3 inverted papilloma; left cluster showed 20 tumours, 18 ITAC, 11 inverted papilloma, 14 inflammatory polyps and 17 normal cases. [file 12672_2026_4508_MOESM2_ESM.tiff]
