# Supplementary material for: DNA methylation analysis with nasal brushing for early diagnosis of sinonasal malignant tumours
Source: Discov Oncol. 2026 Jan 29;17:357. doi: 10.1007/s12672-026-04508-0 (PMC12923719; doi:10.1007/s12672-026-04508-0)
Supplement: Supplementary file 3 — Supplementary Material 3. Risk ratio (RR) and 95% CIs of obtaining a positive methylation score compared to normal mucosa. [file 12672_2026_4508_MOESM3_ESM.pdf]

SupplementaryFile3:

Risk Ratio(RR) and 95% confidence intervals (CI) of obtaining a positive methylation score compared to normal mucosa. P-value derived from Fisher's exact test for the SNIP vs normal comparison. All other RR values are referenced to the normal group. A RR > 1 indicates increased likelihood of score positivity relative to normal mucosa.

		P-value F-Test	RR	L95%CI	U95%CI
**SNIP**			8.19	4.15	16.14
**Inf. Polyp**			2.72	1.18	6.27
**ITAC**	**Vs N**	<0.0001	8,21	4.22	15.98
**SNCC**			6.82	3.44	13.54
